# Consensus molecular subtype 4 (CMS4)-targeted therapy in primary colon cancer: A proof-of-concept study

**DOI:** 10.3389/fonc.2022.969855

**Published:** 2022-09-06

**Authors:** Niek A. Peters, Alexander Constantinides, Inge Ubink, Joyce van Kuik, Haiko J. Bloemendal, Joyce M. van Dodewaard, Menno A. Brink, Thijs P. Schwartz, Martijn P.J.K. Lolkema, Miangela M. Lacle, Leon M. Moons, Joost Geesing, Wilhelmina M.U. van Grevenstein, Jeanine M. L. Roodhart, Miriam Koopman, Sjoerd G. Elias, Inne H.M. Borel Rinkes, Onno Kranenburg

**Affiliations:** ^1^Lab Translational Oncology, Division of Imaging and Cancer, University Medical Center Utrecht, Utrecht University, Utrecht, Netherlands; ^2^Department of Pathology, University Medical Center Utrecht, Utrecht University, Utrecht, Netherlands; ^3^Department of Internal Medicine, Meander Medical Center, Amersfoort, Netherlands; ^4^Department of Internal Medicine/Oncology, Radboud University Medical Center Nijmegen, Nijmegen, Netherlands; ^5^Department of Gastroenterology, Meander Medical Center, Amersfoort, Netherlands; ^6^Department of Medical Oncology, Erasmus Medical Center, Rotterdam, Netherlands; ^7^Department of Gastroenterology, University Medical Center Utrecht, Utrecht University, Utrecht, Netherlands; ^8^Department of Gastroenterology, Diakonessenhuis, Utrecht, Netherlands; ^9^Department of Surgical Oncology, Division of Imaging and Cancer, University Medical Center Utrecht, Utrecht University, Utrecht, Netherlands; ^10^Department of Medical Oncology, University Medical Center Utrecht, Utrecht University, Utrecht, Netherlands; ^11^Julius Centre for Health Sciences and Primary Care, University Medical Center Utrecht, Utrecht University, Utrecht, Netherlands

**Keywords:** colorectal cancer, consensus molecular subtype 4, imatinib, ImPACCT, platelet-derived growth factor receptor (PDGFR)

## Abstract

**Background:**

Mesenchymal Consensus Molecular Subtype 4 (CMS4) colon cancer is associated with poor prognosis and therapy resistance. In this proof-of-concept study, we assessed whether a rationally chosen drug could mitigate the distinguishing molecular features of primary CMS4 colon cancer.

**Methods:**

In the ImPACCT trial, informed consent was obtained for molecular subtyping at initial diagnosis of colon cancer using a validated RT-qPCR CMS4-test on three biopsies per tumor (Phase-1, n=69 patients), and for neoadjuvant CMS4-targeting therapy with imatinib (Phase-2, n=5). Pre- and post-treatment tumor biopsies were analyzed by RNA-sequencing and immunohistochemistry. Imatinib-induced gene expression changes were associated with molecular subtypes and survival in an independent cohort of 3232 primary colon cancer.

**Results:**

The CMS4-test classified 52/172 biopsies as CMS4 (30%). Five patients consented to imatinib treatment prior to surgery, yielding 15 pre- and 15 post-treatment samples for molecular analysis. Imatinib treatment caused significant suppression of mesenchymal genes and upregulation of genes encoding epithelial junctions. The gene expression changes induced by imatinib were associated with improved survival and a shift from CMS4 to CMS2.

**Conclusion:**

Imatinib may have value as a CMS-switching drug in primary colon cancer and induces a gene expression program that is associated with improved survival.

## Introduction

Large-scale gene expression profiling of colon cancer has identified recurrent patterns of gene expression that form the basis for ‘molecular subtyping’. The Consensus Molecular Subtype (CMS) classification system distinguishes four subtypes (CMS1-4) that differ in prognosis and response to systemic therapy (1–4). The gene expression programs that distinguish CMS1-4 also provide opportunities for developing CMS-specific targeted therapies. CMS4 tumors have the highest propensity for developing distant metastases (1), and are characterized by a high content of stromal fibroblasts and, consequently, high expression of mesenchymal genes (5, 6). Candidate molecules for developing CMS4-targeted therapy include the receptor tyrosine kinases (RTKs) Platelet-Derived Growth Factor Receptor alpha and beta (PDGFRA, PDGFRB) and c-KIT (7–9). Indeed, a 4-gene RT-qPCR diagnostic test, which measures PDGFRA, PDGFRB, PDGFC, and KIT, identifies CMS4 CRC with very high sensitivity and specificity (9). Small molecule inhibitors of PDGFR/KIT-family RTKs (e.g. imatinib) are routinely being used for the treatment of gastrointestinal stromal tumors (GIST) and some leukemias, but not CRC (10). In pre-clinical studies, inhibition of PDGFR/KIT signaling reduces tumor cell invasion and metastatic potential in models of mesenchymal-like CRC (7, 8, 11) and other cancer types (12–14), suggesting that imatinib may have value as a CMS4-targeting drug.

The development of CMS4-targeting therapeutic strategies is complicated by intra-tumor CMS heterogeneity. Indeed, many colon tumors consist of CMS4 and non-CMS4 regions (9, 15, 16). Moreover, primary tumors and paired metastases are frequently classified into discordant CMSs (17–19). In addition, radiotherapy and/or chemotherapy may cause a shift in CMS classification (17). To validate the concept of CMS4-targeted therapy, we designed a clinical study in treatment-naïve patients with primary non-metastatic CMS4 colon cancer (ImPACCT; NCT02685046) (20). The aim of ImPACCT was to deliver proof-of-concept that a rationally chosen CMS4-targeting drug has the potential to alter the distinguishing molecular features that are associated with CMS4 colon cancer. Imatinib was selected as a CMS4-targeting drug, based on the very high expression of its targets in CMS4 (this study), its anti-metastatic activity in various pre-clinical models (8, 11, 21), and the potential for rapid future clinical development (22, 23). Comparative analysis of pre- and post-treatment tumor tissue allowed us to assess the effect of imatinib treatment on primary CMS4 colon cancer, and to correlate imatinib-induced gene expression changes with CMS distribution and survival in an independent large colon cancer cohort (1).

## Materials and methods

### Identification of Imatinib as a candidate CMS4-targeting drug

For the identification of potential therapeutic targets in CMS4 CRC we made use of two independent CRC cohorts [GSE3958215 (24) and TCGA16 (25)] and correlated the CMS4-identifying genes from the random forest classifier with the human kinome (p <e-6). Next, we made use of a publicly available database of kinase inhibitors and their quantitative dissociation constants (Kd) for a large panel of human kinases (26). The inhibitors targeting CMS4 tyrosine kinases were than ranked according to their selectivity scores, defined as the number of kinase hits with a Kd<3 µM divided by the number of kinases tested.

### ImPACCT study

The ImPACCT study (NCT02685046) (20) was approved by the medical ethical committee of the University Medical Center Utrecht, the Netherlands (15/527) and the Central Committee on Research Involving Human Subjects (NL50620.041.15). The study was divided into two phases: (i) the biopsy classification phase and (ii) the imatinib treatment phase.

In the first phase, subjects scheduled for a diagnostic colonoscopy on account of either clinical suspicion of CRC or in accordance with the national colorectal cancer population screening program were approached for permission to obtain five additional endoscopic biopsies. Out of these 5 biopsies, 3 were used for RNA isolation and RT-qPCR CMS4 classification. From the day of biopsy acquisition, it took 3-5 days to complete the procedure and provide a multi-region-based molecular classification.

In the second phase, patients diagnosed with CMS4 colon or rectal cancer were approached again for informed consent to receive neo-adjuvant imatinib treatment.

### Patients and samples

Three hospitals (the University Medical Center Utrecht, the Meander Hospital Amersfoort, and the Diakonessenhuis Utrecht) were opened as inclusion centers. Biopsy samples were collected in individual sterile cryotubes and snap-frozen in liquid nitrogen as soon as possible, mostly at the end of the endoscopy procedure. Samples were transported on dry ice and stored at -80°C until further downstream processing.

Patients diagnosed with CMS4 CRC were approached again for informed consent and screening for eligibility for the second phase of ImPACCT, consisting of imatinib treatment. In- and exclusion criteria are provided in **Supplementary Table S1**. In brief, patients were required to (i) be scheduled for surgery for removal of the primary tumor, (ii) not receive any neoadjuvant therapy, (iii) be in good condition (ECOG Performance Status 0 or 1), and (iv) not have metastatic disease. After a consideration period, written informed consent was obtained for treatment with imatinib. Imatinib was administered orally at a daily dosage of 400 mg for 14 days prior to planned tumour resection.

The study was powered for 27 patients to be treated with imatinib (20). The study was terminated after inclusion of 5 patients for preoperative imatinib therapy due to low accrual rate. All patients included in this analysis gave written informed consent. Clinical data were collected from electronic patient records.

### Random forest CMS classification

The random forest CMS classification based on next generation RNA sequencing data was performed as previously described and according to Guinney et al. (1, 9) and can be considered the gold standard. In short, the sequencing libraries were normalized using size-factor normalization using the DESeq2 package (version 3.14) (27) on Bioconductor for R. As the ImPACCT cohort should only consists of CMS4 tumors and the random forest classifier requires a balanced dataset with all subtypes present, we made use of a ‘piggyback’ dataset of 199 primary CRCs and pooled these with the ImPACCT dataset to perform the CMS classification. To this end, we applied the 273-gene random forest CMS classifier (available at Github, https://github.com/Sage-Bionetworks/crcsc; applied with predict.randomForest, R package randomForest version 4.6-10), obtaining for each sample a predicted probability of belonging to one of the CMS subtypes.

### Differential gene expression analysis

For the transcriptomic and principal component analyses, we made use of the R2: Genomics Analysis and Visualization Platform (http://r2.amc.nl). Gene set enrichment analyses were performed using the MSigDB hallmark genesets collection (n=50) (28) and the immune compendium signature collection (29). Differentially expressed signatures were identified using ANOVA and significance levels were corrected using the Bonferoni-method (p-value cutoff ≤0.001).

Differentially expressed genes between pre- and post-imatinib samples were identified using ANOVA with multiple comparison correction using FDR p ≤ 0.001. For the comparison between pre- and post-imatinib biopsies the following signatures were used: 4-gene CMS4 test signature, Desmosome, circulating cell cluster (30), WNT (31), KEGG adherens junctions (32), KEGG cell cycle (32), MSigDB hallmark MYC targets v1 and MTORC1 signaling (28), mTOR TOP targets (33), and CMS4 upregulated genes (1) (**Supplementary Table S2**).

### Statistical analysis

Statistical analysis was performed using GraphPad Prism (software version 9) or R version 4.1.1 for Mac (R Foundation for Statistical Computing, Vienna, Austria; http://www.R-project.org). Differential gene expression and principal component analyses were performed in the R2 Genomics platform (http://r2.amc.nl). To study imatinib induced transcriptional changes in the context of CMS subtypes, we used an independent publicly available large CRC cohort of n = 3232 patients [CMS-3232 (1)]. Differentially upregulated genes after imatinib therapy were used to cluster the CMS-3232 cohort into a low- and high-expression subgroup using the k-means cluster algorithm. For comparisons between pre- and post-imatinib biopsies a linear mixed model was used to account for clustering effects within patients using a random intercept per patient. Associations between continuous variables were assessed using marginal Pearson correlation coefficients for clustered data (34). Data were compared using two-sided Pearson χ^2^, Fisher’s exact, Student’s t, or Mann-Whitney-U-test as appropriate. Results with p-values smaller than 0.05 were considered statistically significant (*). All statistical tests performed in this study were two-sided. P-values smaller than 0.01, 0.001, and 0.0001 are indicated by (**), (***), and (****), respectively.

## Results

### Identification of imatinib as a candidate CMS4-targeting drug

To search for potential therapeutic targets in CMS4 CRC we focused on the human kinome. Expression of 55 kinases (22 tyrosine kinases (TK)) was positively correlated with expression of CMS4 signature genes in two independent colon cancer cohorts (p<e-6; **Supplementary Table S3**). A large number of TK inhibitors (TKIs) are approved and available for the treatment of cancer and other diseases. By applying a dataset of quantitative dissociation constants (Kd) of drug-target interactions for 72 distinct kinase inhibitors (26) a list of candidate CMS4-targeting TKIs was identified containing six FDA-approved anti-cancer drugs (**Supplementary Table S4**). Ranked from high-to-low selectivity for inhibiting CMS4 TKs, these are imatinib, nilotinib, sorafenib, pazopanib, vandetanib, and dasatinib (**Supplementary Table S4**; **Supplementary Figure S1**). None of these TKIs are currently indicated for the treatment of colon or rectal cancer. Of the 6 candidate CMS4-targeting drugs, imatinib was the most selective, targeting PDGFRA, PDGFRB, KIT and the collagen receptor DDR2 in the lower nanomolar range (**Supplementary Table S5**). Based on this analysis, the available toxicity data, and the reported anti-tumorigenic and anti-metastatic activity in pre-clinical colon cancer models (8, 11, 21–23), imatinib was chosen as CMS4-targeting drug in ImPACCT.

### Selection of patients with CMS4 colon cancer at initial diagnosis

**Figure 1:**
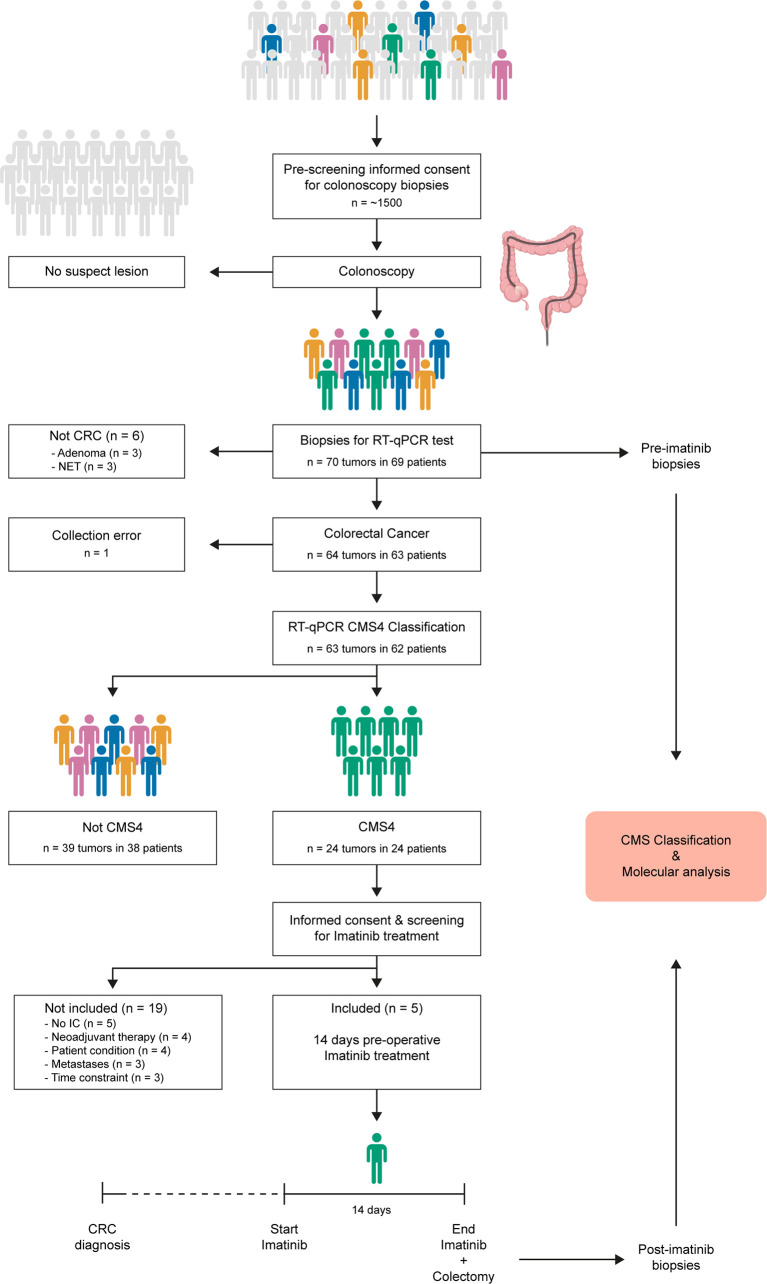
ImPACCT study flowchart. Individuals scheduled for a colonoscopy procedure were approached to obtain informed consent for acquisition of 5 additional biopsies for CMS4 testing in case suspect lesions were found, and for approval to approach them again in case the tumor was diagnosed as CMS4. Patients with CMS4 CRC were approached to obtain informed consent for the second part of the study (imatinib treatment), and were screened for eligibility. Five patients received imatinib treatment for 14 days prior to surgery. Pre-treatment diagnostic biopsies and post-treatment biopsies from the resected primary tumors were used for CMS classification and additional molecular analyses.CMS, consensus molecular subtype; RT-qPCR, real-time quantitative polymerase chain reaction; IC, informed consent; CRC, colorectal cancer.

Between August 2016 and August 2019, approximately 1500 individuals who were scheduled for colonoscopy were approached for informed consent for the acquisition of additional biopsies for molecular diagnosis using a validated 4-gene RT-qPCR CMS4 test (9) (**Figure 1**). In total, 350 endoscopic biopsies were collected from 70 tumors from 69 patients (**Figure 1** and **Supplementary Table S6**). Of these, 63 tumors from 62 patients were histologically confirmed colorectal cancer (58 colon; 5 rectum) and used for CMS4 testing (**Table 1**).

**Table 1:** Characteristics of colorectal cancer patients biopsied for CMS4 identification.

Characteristic	Not CMS4, N = 39^1^	CMS4, N = 24^1^	p–value^2^
Age at diagnosis	74 (52–86)	62 (48–83)	<0.001
Sex			0.35
Male	22 (58)	11 (46)	
Female	16 (42)	13 (54)	
Diagnosis			>0.99
Colorectal Cancer	39 (100)	24 (100)	
Adenoma	0 (0)	0 (0)	
Neuroendocrine tumor	0 (0)	0 (0)	
Histology			0.29
Adenocarcinoma	25 (68)	21 (91)	
Mucinous adenocarcinoma	8 (22)	2 (8.7)	
Signet–ring cell	2 (5.4)	0 (0)	
Other	1 (2.7)	0 (0)	
Not reported	1 (2.7)	0 (0)	
Sidedness			0.003
Right colon	26 (67)	6 (25)	
Left colon	11 (28)	15 (62)	
Rectum	2 (5.1)	3 (12)	
AJCC TNM Stage			0.62
1	7 (19)	3 (12)	
2	12 (32)	5 (21)	
3	15 (41)	13 (54)	
4	3 (8.1)	3 (12)	
Differentiation			>0.99
Well	0 (0)	0 (0)	
Well–moderate	28 (88)	21 (91)	
Moderate	2 (6.2)	1 (4.3)	
Poor	0 (0)	0 (0)	
Undifferentiated	2 (6.2)	1 (4.3)	
MSI			0.047
MSS	18 (62)	18 (90)	
MSI	11 (38)	2 (10)	
Heterogeneity in CMS4 status of biopsies	6 (15)	14 (58)	<0.001
(Serious) Adverse Event after colonoscopy	0 (0)	1 (4.2)	0.39
Underwent surgery	33 (89)	23 (96)	0.64
Procedure			<0.001
Abdominoperineal Resection	0 (0)	1 (4.3)	
Extended Hemicolectomy	4 (12)	0 (0)	
Hemicolectomy Left	2 (6.2)	3 (13)	
Hemicolectomy Right	16 (50)	5 (22)	
Low Anterior Resection	0 (0)	9 (39)	
Sigmoid Resection	8 (25)	5 (22)	
Transverse Colectomy	2 (6.2)	0 (0)	
Laparoscopic			0.34
Open	3 (9.4)	5 (22)	
Laparoscopic	23 (72)	14 (61)	
Laparoscopic converted to open	6 (19)	3 (13)	
Robot	0 (0)	1 (4.3)	
Primary anastomosis	32 (100)	17 (74)	0.003
Length of stay (days)	5.5 (2.0–38.0)	5.0 (3.0–42.0)	0.92
Complications Clavien Dindo >II	14 (42)	8 (35)	0.56
90–day mortality	0 (0)	1 (4.3)	0.43
Pre–operative Imatinib Therapy	0 (0)	5 (21)	n.a.

^1^Median (Minimum–Maximum), n (%). ^2^Wilcoxon rank sum test, Pearson’s Chi–squared test, Fisher’s exact test, where appropriate. n.a., not applicable.

**Figure 2:**
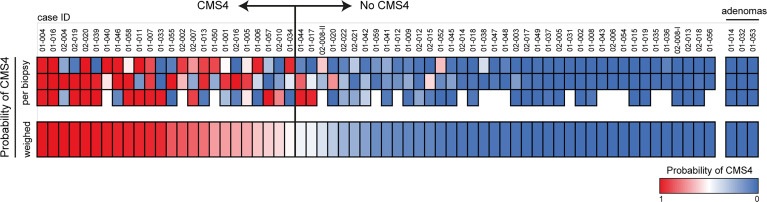
. CMS4 assessment on diagnostic biopsies and patient selection. Diagnostic biopsies (3 per tumor) were processed for RNA isolation and subsequent CMS4 testing, using a previously designed and validated RT-qPCR test (9). The heatmap shows CMS4 probabilities per-biopsy (top) and the weighed mean probabilities per tumor, to account for intra-tumor CMS4 heterogeneity. If the weighed mean probability was higher than 50%, tumors were classified as CMS4 (n=24) and the patients were approached for the second part of the study. The cohort contained 3 histologically confirmed adenomas (right sub-panel).

To account for intra-tumor CMS heterogeneity RNA was isolated from 195 biopsies from 63 tumors. This yielded 172 RNA samples of sufficient quantity and quality for subsequent CMS4 testing. Of these 172 biopsies, 52 (30%) were classified as CMS4 (**Figure 2**). Different regions from the majority of individual tumors displayed considerable variation in CMS4 probability (**Figure 2**). After calculating the weighed mean CMS4 probability of 2-3 biopsies per tumor, 24 of the 63 evaluable tumors (38%) were classified as CMS4 (**Figure 2**; **Table 1**). CMS4 tumors were diagnosed significantly more frequently in younger patients (< 0.001) and in left-sided colon cancers (p<0.001) (**Table 1**). Moreover, micro-satellite instability (MSI) was detected significantly less frequently in CMS4 versus non-CMS4 tumors (2/20 vs. 11/29; p=0.047). The fraction of CMS4 tumors increased with higher TNM stage (**Supplementary Figure S2**). Although this trend did not reach statistical significance, it is in line with a previous report (35). All 9 biopsies obtained from three adenomas were classified as non-CMS4 (**Figure 2**).

### Neoadjuvant imatinib therapy

All patients with CMS4 tumors were approached to obtain informed consent for inclusion in the second phase of the study: two weeks preoperative imatinib treatment. Of the 24 patients whose primary tumors were classified as CMS4 at first diagnosis, 14 were ineligible for the second part of the study, because i) they received neoadjuvant chemotherapy (n=4), ii) the patients were in poor condition (n=4), iii) distant metastases were detected (n=3), or iv) the preoperative window was shorter than 2 weeks (n=3). Of the remaining 10 patients with primary CMS4 CRC, 5 patients (all colon cancer) consented to participate in the second phase of the study and received 14 days of imatinib treatment prior to surgical removal of the primary tumor (**Figure 1**, **Table 2**). No serious adverse events were observed during imatinib therapy. Minor adverse events were observed in 3 patients and included fatigue, edema, eye irritation, nausea, and dizziness.

**Table 2:** Characteristics of CMS4 colon cancer patients who received neoadjuvant imatinib therapy.

Characteristic	N = 5^1^
Age at diagnosis, No. (%)	56.0 (48.0–62.0)
Sex	
Male	3 (60)
Female	2 (40)
Height (cm), median (min–max)	178.0 (164.0–183.0)
Weight (kg), median (min–max)	95 (76–126)
ECOG status, No. (%)	
WHO 0	3 (60)
WHO 1	2 (40)
Location, No. (%)	
Caecum	1 (20)
Sigmoid	3 (60)
Transverse colon	1 (20)
Tumor stage, No. (%)	
T3	4 (80)
T4	1 (20)
Nodal stage, No. (%)	
N0	1 (20)
N1	2 (40)
N2	2 (40)
Metastatic stage, No. (%)	
M0	5 (100)
AJCC TNM Stage, No. (%)	
2	1 (20)
3	4 (80)
Differentiation, No. (%)	
Moderate	2 (40)
Well	3 (60)
Procedure, No. (%)	
Hemicolectomy Left	1 (20)
Hemicolectomy Right	1 (20)
Low Anterior Resection	2 (40)
Sigmoid Resection	1 (20)
Length of stay (days), median (min–max)	8.0 (4.0–18.0)
Complications Clavien Dindo >II, No. (%)	3 (60)
Adverse Events, No. (%)	3 (60)
Serious Adverse Events, No. (%)	1 (20)
90–day mortality, No. (%)	0 (0)

^1^Median (Minimum–Maximum), n (%)

Peri- and postoperative adverse events were documented in 4 patients and included gastroparesis and stomach ache. One patient underwent an extensive multivisceral resection involving the rectosigmoid, a portion of the vagina, the upper bladder wall, and the adnex. This patient experienced intra-operative haemorrhage which was followed by admission to the intensive care unit (ICU) and an extended postoperative hospital stay. Given the extent of the surgical procedure, the relationship of these adverse events with imatinib treatment is unlikely. Median follow-up time in the CMS4 and non-CMS4 groups were 34.2 months and 37.1 months respectively. 2-year overall-survival (OS) in the CMS4 and non-CMS4 groups were 86.7% (95%-CI, 73.8% - 100%) and 71.7% (95%-CI, 58.2% - 88.3%) respectively. Kaplan Meier survival estimates were not significantly different between the 2 groups (p=0.29; **Supplementary Figure S3**). Within the CMS4 group, overall survival between imatinib-treated and untreated patients was not significantly different (**Supplementary Figure S3**).

### Imatinib treatment shifts CMS4 tumors to a more epithelial phenotype

**Figure 3:**
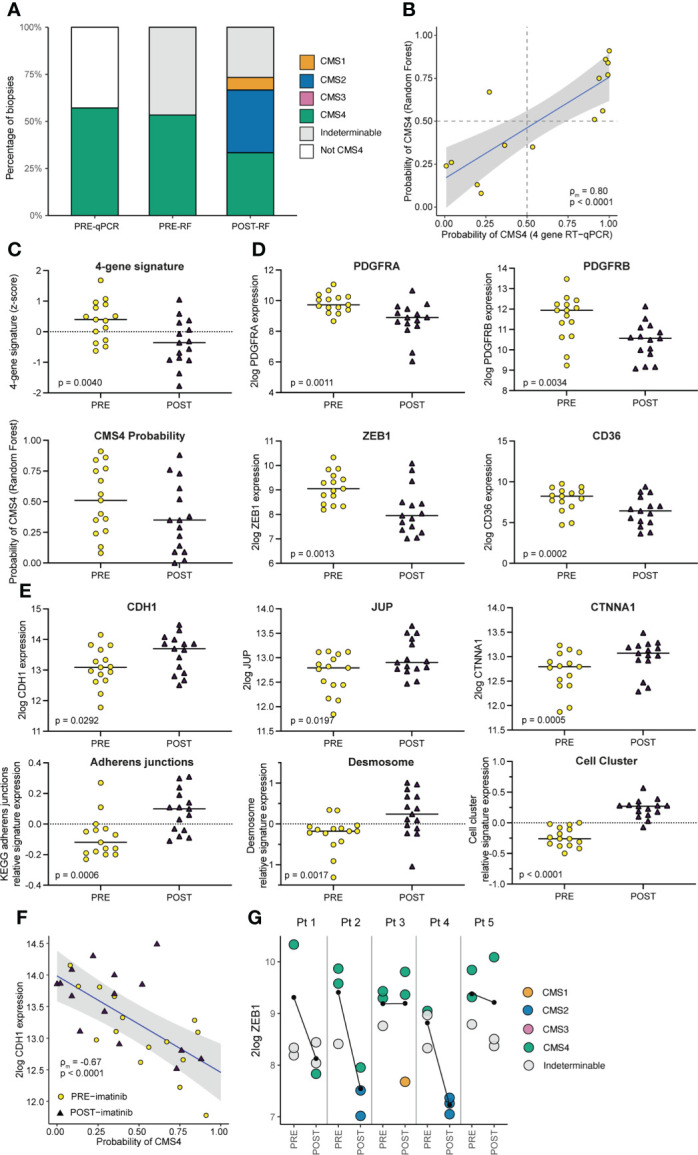
Imatinib treatment of primary CMS4 CRC results in a mesenchymal-to-epithelial phenotype shift. **(A)** Bar graph summarizing CMS classification of tumor tissue samples PRE and POST imatinib treatment, measured by the RT-qPCR test and the CMS random forest (RF) classifier applied to RNA sequencing data. **(B)** XY-plot showing the correlation between CMS4 probabilities of pre-treatment diagnostic biopsies as measured by the RT-qPCR test and the RF classifier. ρ_m_ denotes the marginal Pearson correlation coefficient for clustered data (34) with two-sided p-value. **(C)** Dot-plots showing expression (mean z-scores) of a signature comprised of the 4 genes in the CMS4 test (*PDGFRA, PDGFRB, PDGFC, KIT*) and the CMS4 probabilities generated by the RF classifier, in tissue samples PRE and POST imatinib treatment. P values were generated using ANOVA and a linear mixed model. **(D)** Dot plots showing 2log expression levels of *PDGFRA*, *PDGFRB*, *ZEB1*, and *CD36* in tissue samples PRE and POST imatinib treatment. P values were generated using a two-sided Student’s *t*-test. **(E)** Dot plots Graphs showing 2log expression values of epithelial junction genes (*CDH1*, *JUP*, and *CTNNA)* and expression of signatures for Adherens Junctions, Desmosomes, and genes upregulated in epithelial cell clusters versus single cells in tissue samples PRE and POST imatinib treatment. P values were generated using ANOVA and a linear mixed on pre- vs post-treatment biopsies. **(F)** XY-plot showing the (negative) correlation between *CDH1* expression and CMS4 probabilities (RF) in tissue samples PRE and POST imatinib treatment. ρ_m_ denotes the marginal Pearson correlation coefficient for clustered data with two-sided p-value. **(G)** Dot plot showing *ZEB1* expression in tissue samples PRE and POST imatinib treatment in individual patients with color-coded CMS classification. The black lines indicate the change in mean *ZEB1* expression following imatinib treatment.

Pre-treatment biopsies (obtained during diagnostic colonoscopy) and post-treatment biopsies (obtained from the surgical resection specimen) were processed for RNA isolation and RNA sequencing. Molecular subtyping of the samples by applying the random forest CMS classifier revealed that pre-treatment samples were classified either as CMS4, or as indeterminable (if the probability of none of the subtypes was more than 0.5) (**Figure 3A**). The CMS4 probabilities of the pre-treatment samples showed a strong and significant correlation with the CMS4 scores that were generated by the CMS4 4-gene RT-qPCR test (ρ_m_=0.80, p<0.0001; **Figure 3B**). Of the 15 biopsies analysed prior to treatment none were classified as CMS1-3. However, after imatinib treatment 6 of the 15 biopsies (40%) were classified as CMS1 (n=2) or CMS2 (n=4), while the incidence of CMS4 biopsies was reduced from 55% to 33% (**Figure 3A**). Overall, imatinib treatment caused a reduction of the average CMS4 probability, although this was not statistically significant (**Figure 3C**). However, we did observe a significantly reduced expression of the 4 CMS4-identfying genes in the RT qPCR test that was used to include the patients (**Figure 3C**). Moreover, expression of specific mesenchymal genes such as ZEB1, PDGFRA, PDGFRB, and CD36 was strongly and significantly reduced after imatinib treatment (**Figure 3D**). Epithelial cell-cell contacts are mediated by adherens junctions, desmosomes and tight junctions. Expression of *CDH1* and *CTNNA1*, encoding the adherens junction components E-cadherin and alpha-catenin, and an adherens junction signature, was significantly increased following imatinib treatment (**Figure 3E**). Likewise, expression of the desmosome gene *JUP*, encoding Plakoglobin, and a desmosome signature was also significantly higher following imatinib treatment (**Figure 3E**). Moreover, an independent signature distinguishing circulating tumor cell clusters from single cells, was also significantly higher in post-treatment samples (**Figure 3E**). By contrast, expression of genes encoding tight junction proteins did not change following imatinib treatment. Expression of CDH1 was inversely correlated with CMS4 probability (**Figure 3F**). Expression of the key EMT-driving transcription factor ZEB1 was strongly reduced following imatinib treatment in 3/5 patients (**Figure 3G**), similar to what we have previously observed in pre-clinical CRC models (8). Biopsies with the highest ZEB1 expression were mostly derived from pre-treatment tumors (8/11; 73%), while biopsies with the lowest ZEB1 expression were all derived from post-treatment tumors (9/9; 100%) (**Figure 3G**).

**Figure 4:**
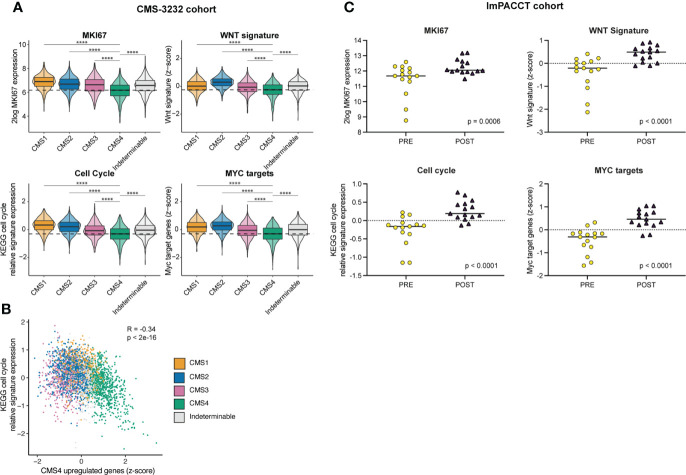
Imatinib treatment of primary CMS4 CRC causes increased expression of proliferation-associated genes. **(A)** Tukey box and violin plots showing expression of the proliferation marker *MKI6*7 and signatures reflecting cell cycle activity (KEGG), WNT target genes (31), and MYC target genes (39) in CMS1–4 in the CMS–3232 cohort. Statistically significant differences were identified using one–way analysis of variance (ANOVA) with subsequent *post–hoc* pairwise comparisons using *t*–tests with pooled SD using Bonferroni multiple comparison p–value adjustment. **(B)** XY–plot demonstrating the (negative) correlation between CMS4–identfying genes in the RF classifier and the KEGG pathway signature genes reflecting cell cycle activity. R denotes the Pearson correlation coefficient with two–sided p–value in the CMS–3232 cohort. CMS1–4 are color–coded. **(C)** As in **(A)** but in the ImPACCT cohort. Statistically significant differences were identified using two–sided ANOVA and a linear mixed model.

Mesenchymal tumor phenotypes are generally accompanied by reduced proliferation. Indeed, high expression of proliferation signatures and Wnt target genes are associated with good prognosis and reduced metastatic capacity in CRC (36–38). To further elucidate this observation in the context of CMS subtypes in CRC, we made use of a publicly available dataset of 3232 primary colon cancers (CMS-3232). In line with this notion, we found that expression of the generic proliferation marker MKI67, WNT- and MYC-target genes, and the KEGG pathway ‘cell-cycle’ are expressed at significantly lower levels in CMS4 than in any of the other subtypes in primary colon cancer (**Figure 4A**). Moreover, expression of CMS4-identifying genes from the random forest classifier were inversely correlated to expression of genes in the KEGG pathway ‘cell-cycle’ (R = -0.34, p<2e-16; **Figure 4B**). In ImPACCT, imatinib treatment of CMS4 tumors caused a significant increase in the expression of MKI67, WNT- and MYC-target genes, and KEGG pathway cell cycle genes (**Figure 4C**).

### Imatinib induces a gene expression program that is associated with improved prognosis

**Figure 5:**
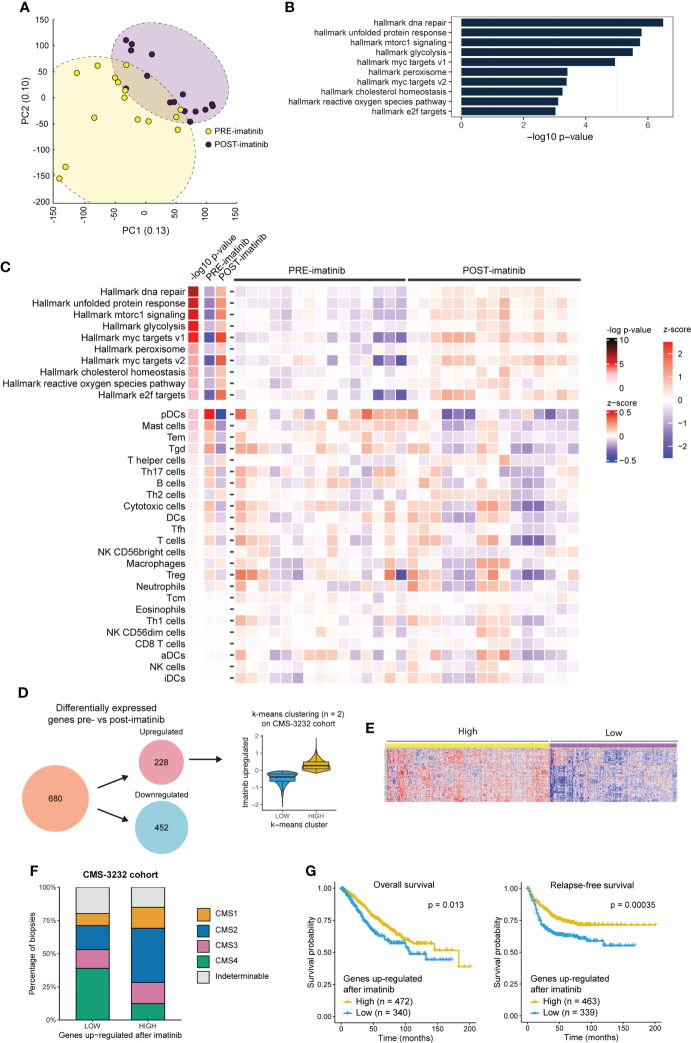
Imatinib treatment of primary CMS4 CRC induces a phenotype that is associated with better prognosis. **(A)** Principle component analysis based on expression of all genes. PRE and POST imatinib samples are color–coded. **(B)** Bar plot showing the significant up– and down–regulated cancer hallmark signatures (40) (n = 10/50) between pre– and post–imatinib biopsies ranked according to significance (min–log10 p–values). **(C)** Heatmap showing expression of the 10 significantly upregulated hallmark signatures and a compendium of immune signatures (29) in PRE and POST imatinib treatment samples. **(D)** Differential gene expression analysis (ANOVA FDR p ≤ 0.001) identified 680 differentially expressed genes of which 228 were up– and 452 were down–regulated after imatinib therapy. The 228 imatinib–induced genes were then used to cluster the CMS3232 cohort (1) into LOW and HIGH expression subgroups using the k–means algorithm. **(E)** Heatmap showing expression of imatinib–induced genes in the LOW and HIGH expression subgroups. **(F)** Stacked barplot showing the CMS distribution in subgroups of tumors expressing LOW and HIGH levels of imatinib–induced genes. **(G)** Kaplan Meier curves showing overall (left) and relapse–free (right) survival in subgroups of stage II–III tumors in the CMS3232 cohort (1) expressing LOW and HIGH levels of imatinib–induced genes. A two–sided log–rank test was applied to assess the significance of the survival differences between the two groups.

To explore the transcriptomic effects of imatinib treatment in an unbiased fashion, the RNAseq data were subjected to a dimensionality reduction analysis (principal component analysis; PCA). Interestingly, the samples segregated according to treatment status (pre-versus post-treatment) indicating that imatinib therapy had a major effect on global gene expression patterns (**Figure 5A**). Furthermore, imatinib treatment resulted in significantly increased expression of 10 signatures reflecting specific Cancer Hallmarks (Molecular Signature Database [MSigDB) (39)], including ‘mTORC1 signaling’ and ‘E2F targets’ (Figure 5B, **Supplementary Figure S4**). Of note, these pathways have previously been linked to a mesenchymal-to-epithelial (i.e. invasive-to-proliferative) phenotype shift (40).

Next, we analysed whether imatinib treatment altered the immune landscape of CRC. To this end, we made use of the immune compendium signature collection (29) and found that imatinib treatment did not significantly alter expression of immune-related gene signatures (**Figure 5C**).

Differential gene expression analysis between pre- and post-treatment biopsies identified 228 significantly upregulated genes following imatinib treatment, and 452 downregulated genes (FDR<0.001; **Supplementary Table S7**). To assess the potential prognostic value of this shift in ‘molecular phenotype’ we made use of the CMS-3232 primary CRC cohort with annotated CMS status and survival data (1). The 228 genes upregulated after imatinib treatment were used to cluster the Stage II and III tumors in this cohort into low and high expression subgroups using the k-means algorithm (**Figure 5D**). The corresponding heatmap (**Figure 5E**) shows that the genes induced by imatinib are strongly co-regulated in primary CRC. Analysis of the CMS distribution in subgroups expressing high versus low levels of imatinib-induced genes revealed a significantly lower proportion of CMS4 tumors in the high expression subgroup (12% vs. 39%; p < 2.2e-16; **Figure 5F**). Moreover, relapse-free and overall survival were significantly better in the subgroup expressing high levels of imatinib-induced genes (**Figure 5G**). Overall, the data suggest that neo-adjuvant imatinib treatment causes a phenotypic (mesenchymal-to-epithelial) shift that is associated with better survival.

### Imatinib alters mTORC1 signaling

**Figure 6:**
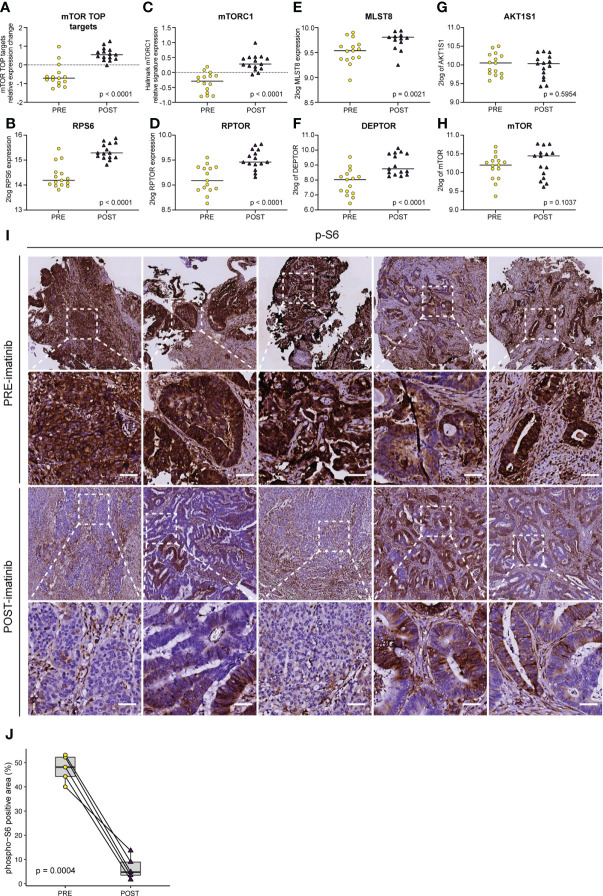
Imatinib inhibits ribosomal protein S6 phosphorylation and causes transcriptional activation of the mTORC1 pathway. Expression levels of **(A)** mTORC1 TOP target mRNAs (33), **(B)** ribosomal protein S6 (*RPS6*), **(C)** the Hallmark mTORC1 signature, and the individual mTORC1 components **(D)** *RPTOR*, **(E)** *MLST8*, **(F)** *DEPTOR*, **(G)** *AKT1S1*, and **(H)** *MTOR.* Statistically significant expression differences were identified using ANOVA and a linear mixed model. **(I)** Immunohistochemistry (IHC) for the detection of phosphorylated ribosomal protein S6 (pS6) on PRE–treatment (upper row) and POST–treatment (lower row) biopsies. Representative images of the stained sections are shown. Scale bar, 50 μm **(J)** QuPath software (41) was used to quantify the pS6 IHC signal in the epithelial compartment in pre– and post–imatinib biopsies. Values were then plotted in Tukey boxplots and the significance of the observed staining difference was assessed using a two–sided paired Student’s *t*–test.

One of the cancer hallmark pathways that was most significantly upregulated in imatinib-treated tumors was ‘mTORC1 signalling’ (**Figures 5B, C**). The mTORC1 protein complex plays an essential role in the translation of mRNAs containing a terminal oligo-pyrimidine (TOP) motif, which mainly encode translational initiation and elongation factors and ribosomal proteins necessary for cell growth and proliferation (33). Imatinib treatment significantly increased the expression of virtually all known TOP mRNA mTORC1 targets, as well as expression of three of the five mTORC1 complex subunits (MLST8, DEPTOR, RPTOR) (**Figures 6A–F**). Expression of the other two mTORC1 subunits was unaltered (MTOR, AKT1S1) (**Figures 6G, H**). Some of the best characterized substrates for mTORC1 are the ribosomal protein S6 kinases (S6K1 and S6K2) which phosphorylate ribosomal protein 6 (RPS6) and multiple other substrates to control protein translation, cell size and cell survival (42). Importantly, mTORC1 activation of S6K is essential for maintaining proliferation of APC-deficient intestinal adenomas in mice (43). The observed upregulation of mTORC1-encoding genes and the Hallmark mTORC1 pathway suggests that this pathway may have been activated following imatinib treatment. Therefore, we used antibodies recognizing phosphorylated RPS6 (pS6) as a tool for assessing the activity of the mTORC1-S6K pathway in pre- and post-treatment tumor samples *in situ*. Surprisingly, we found that imatinib caused a profound inhibition of S6 phosphorylation in the tumor cells of all post-treatment samples examined (**Figures 6I–J**).

## Discussion

In this study we have provided proof-of-concept that the aggressive phenotype of CMS4 CRC can be mitigated by rationally chosen targeted therapy. The mesenchymal-to-epithelial phenotype shift following imatinib therapy coincided with increased expression of WNT- and MYC-target genes and signatures reflecting proliferation. Accelerated proliferation may – at first sight – not be considered a desired effect of any anti-cancer therapy. However, high expression of proliferation signatures and WNT target genes are associated with good prognosis and reduced metastatic capacity in CRC (36–38). Proliferation and invasion are often inversely regulated in tumor biology, supporting the notion that proliferating tumor cells have to switch their transcriptional state (through EMT) in order to acquire invasive and metastatic properties (40, 44, 45). Proliferating tumor cells require high expression of mTORC1 and its target genes to meet their anabolic demand (46). The high expression of mTORC1 in imatinib-treated tumors may therefore simply reflect the MET phenotype switch. Interestingly, activation of the mTORC1 pathway also plays an important role in acquired resistance to imatinib (47–49). It is therefore possible that the profound inhibition of mTORC1 signaling (*i.e.* reduced S6 phosphorylation) in imatinib-treated tumors has caused activation of a transcriptional feedback program in an attempt to restore pathway activity. Further preclinical work should reveal whether prolonged treatment of CMS4 CRC with imatinib monotherapy indeed leads to re-activation of mTORC1 signaling. Combination treatments consisting of imatinib and mTOR inhibitors are currently being evaluated in clinical trials, although not in colon cancer patients [NCT01275222 and (50)].

Clinical application of the CMS system not only requires the development of effective subtype-targeted therapies, but also the generation of diagnostic tools that allow rapid subtype assessment in routine clinical practice. Several tissue-based diagnostic tools have been developed for clinical CMS stratification (2, 9, 51, 52). However, all methods suffer from the existence of intra-and inter-tumor CMS heterogeneity and, thus, from sampling bias. In the present study we have dealt with this problem by taking a multi-biopsy approach, coupled to a weighing strategy of RT-qPCR test results (9), and have demonstrated the feasibility of identifying primary CMS4 CRC at initial diagnosis on endoscopic biopsies. We have focused on primary colon cancer, because the CMS classification was based on this disease entity (1). However, in patients with metastatic disease, inter- and intra-tumor heterogeneity will pose a more profound problem, simply because tumor load is higher and more diverse, and sampling options are limited. One potential solution would be the design and development of CMS-specific molecular imaging strategies (53).

The ImPACCT study was discontinued due to slow accrual. The major logistical challenge was the requirement to obtain informed consent from every individual prior to colonoscopy. However, only 2-5% of people undergoing a colonoscopy are diagnosed with cancer, and only 25% of these tumors are CMS4 colon cancer. In ImPACCT more than 1.500 people undergoing a colonoscopy had to be approached to ultimately include 5 patients for treatment (0.3%). The inclusion of tissue- or imaging-based molecular subtyping as part of the routine diagnostic workup for primary colon cancer will therefore greatly facilitate future studies developing CMS-targeted therapy.

In conclusion, this study demonstrates the feasibility of mitigating the aggressive biology of CMS4 primary colon cancer with targeted therapy in pre-selected cancer patients. The gene expression changes caused by imatinib treatment were indicative of a mesenchymal-to-epithelial phenotype shift and were associated with better prognosis. A logical next step would be to evaluate whether ‘CMS4-switch-therapy’ can sensitize CMS4 colon cancer to standard chemotherapy regimens.

## Data availability statement

The original contributions presented in the study are publicly available. This data can be found at the Gene Expression Omnibus (https://www.ncbi.nlm.nih.gov/geo/) under the Accession Number GSE3958215.

## Ethics statement

The studies involving human participants were reviewed and approved by Medical Ethics Review Board in the University Medical Center Utrecht. The patients/participants provided their written informed consent to participate in this study.

## Author contributions

Conceptualization: IU, ML, MK, SE, IBR, OK. Data curation: NP, AC, IU, JK, HB, JD, MB, TS, LM, SE, IB, OK. Investigation and experiments: NP, AC, IU, JK. Methodology: SE. Formal analysis: NP, AC, IU, SE, IR, OK. Supervision: HB, JD, JG, IB, OK. Writing – original draft: NP, AC, IU, SE, IB, OK. Writing – review and editing: NP, AC, IU, JK, HB, JD, MB, TS, MPL, ML, LM, JG, WG, JR, MK, SE, IB, OK. All authors contributed to the article and approved the submitted version.

## Funding

This work was supported by grants from the Dutch Cancer Society (UU-2014-6617 to IU) and ZonMw (95104001).

## Conflict of interest

The authors declare that the research was conducted in the absence of any commercial or financial relationships that could be construed as a potential conflict of interest.

## Publisher’s note

All claims expressed in this article are solely those of the authors and do not necessarily represent those of their affiliated organizations, or those of the publisher, the editors and the reviewers. Any product that may be evaluated in this article, or claim that may be made by its manufacturer, is not guaranteed or endorsed by the publisher.
