# Supplementary material for: Consensus molecular subtype 4 (CMS4)-targeted therapy in primary colon cancer: A proof-of-concept study
Source: Front Oncol. 2022 Sep 6;12:969855. doi: 10.3389/fonc.2022.969855 (PMC9486194; doi:10.3389/fonc.2022.969855)
Supplement: Supplementary file 1 [file DataSheet_1.pdf]

Supplementary Materials

Table of contents: **Page**

Supplementary Methods

RNA isolation and RT-qPCR test for CMS4 selection 2
Library preparation and next generation sequencing 2
Immunohistochemistry 2
Histopathological quantification 2
Prognostic relevance of imatinib-induced gene expression changes 3
References 4

Supplementary Tables

Supplementary Table S1 — In- and exclusion criteria for treatment with Imatinib in patients with CMS4 colon cancer in the ImPACCT trial. 5
Supplementary Table S2 — Gene set signatures used in the analyses of the ImPACCT study 6
Supplementary Table S3 — Kinases correlated with CMS4 20
Supplementary Table S4 — Kd values of 72 tyrosine kinase inhibitors for CMS4 related kinases. 24
Supplementary Table S5 — FDA approved tyrosine kinase inhibitors against CMS4 related kinases 27
Supplementary Table S6 — Patient characteristics of all patients who underwent additional research biopsies for a suspect lesion during colonoscopy in the ImPACCT study. 28
Supplementary Table S7 — Differentially expressed genes of pre- vs post-imatinib biopsies in the ImPACCT study 29

Supplementary Figures

Supplementary Figure S1 — Imatinib is a selective inhibitor of tyrosine kinases overexpressed in CMS4 CRC. 45
Supplementary Figure S2 — AJCC TNM stage correlated with CMS4 subtype 46
Supplementary Figure S3 — Overall survival of CRC patients in the ImPACCT study 47
Supplementary Figure S4 — Gene-set enrichment analysis in PRE- vs POST-imatinib biopsies 48

Supplementary Methods

RNA isolation and RT-qPCR test for CMS4 selection

RNA isolation and the 4-gene RT-qPCR test was performed as previously described.[1] Three of the five collected endoscopic biopsies were initially used for RNA isolation. In brief, biopsies were homogenized using the tissue homogenizing CKMix ceramic beads (Precellys, P000918-LYSK0-A) and subsequently processed for RNA isolation using the column-based RNA isolation (RNeasy Kit, Qiagen). RNA yield and quality were evaluated using spectrometry (Nanodrop 2000/200c, Thermo Fisher) and the BioAnalyzer (2100 Bioanalyzer, Agilent), respectively. If the yield and/or quality of two out of three samples was too low (i.e. concentration >20 ng/ul or RIN value < 6), one of the remaining biopsies was used for RNA isolation and CMS classification as well. A minimum of two biopsies with sufficient yield and quality were used for final RT-qPCR CMS4 classification.

cDNA was prepared using the High-Capacity RNA-to-cDNA kit (Applied Biosciences). The reverse transcription (RT) product was used for quantitative real time polymerase chain reaction (qPCR) analysing the following targets: *PDGFRA, PDGFRB, PDGFC, KIT, GAPDH*. ΔCt values of each sample individually served as input for the test algorithm. We mathematically pooled all samples per patient to derive a weighed mean change of CMS4, so that the relative contribution of each of the biopsies to the final test score is determined by the RNA concentration of that sample. The mean ΔCt values are then entered into the 4-gene RT-qPCR prediction test to yield the final weighed mean probability of CMS4. The patient was considered CMS4 positive with a CMS4 probability of ≥0.5.

Library preparation and next generation sequencing

Library preparation and Next Generation Sequencing (NGS) was performed by USEQ (Utrecht Sequencing Facility, Utrecht, The Netherlands). For the generation of sequencing libraries, Truseq RNA strated polyA (Illumina) was used. Libraries were sequenced on the Illumina NextSeq500 platform on the High Output 1 x 75bp configuration. This resulted in a minimal sequencing depth of 23 million reads per sample. RNA sequencing was performed on the same RNA of the pre-imatinib biopsies that was used for CMS4 classification by RT-qPCR. Sequencing data have been made available in the NCBI's Gene Expression Omnibus repository (GEO:)

Immunohistochemistry

For immunohistochemistry the diagnostic colonoscopy biopsies and the diagnostic surgical resection specimens were used, as the research biopsies obtained in the ImPACCT trial are used for molecular analyses. Specimens were formalin fixed and paraffin embedded and cut into 4 µm sections. Slides were deparaffinized by immersing the slides in xylene and subsequently rehydrated in a graded alcohol series (100% ethanol to 70% ethanol). Briefly, after epitope retrieval was carried out in 10 mM Sodium Citrate (pH 6.0), endogenous peroxidase was blocked in a PBS H_2O_2 buffer. Tissue sections were incubated overnight at 4°C using antibodies against phosphorylated S6 (1:200; CST2211, Cell Signaling Technology). Goat-anti-Rb-poly-HRP was used as secondary antibody and signal amplification. All slides were developed with diaminobenzidine (DAB) followed by hematoxylin counterstaining. Slides were air dried and subsequently cover slipped. Stained slides were scanned using a NanoZoomer Digital Slide Scanner (Hamamatsu).

Histopathological quantification

Immunohistochemically stained sections of pre- and post-imatinib treatment biopsies were loaded into QuPath v0.3.0. For each patient (in the pre-treatment and post-treatment condition), representative annotations were created in the epithelial compartment of the tumor with a minimum of n=11 and a maximum of n=86 representative annotations per condition. The image was color deconvoluted across both the DAB and the Hematoxylin staining vectors and subsequently thresholded to calculate a positive staining area percentage for the epithelial compartment. The mean

DAB positive area percentages were calculated for each patient per condition and compared using a two-sided paired Student's *t*-test.

Prognostic relevance of imatinib-induced gene expression changes

To determine the potential prognostic relevance of imatinib-induced gene expression changes, the list of significantly upregulated genes after imatinib treatment in the five treated ImPACCT patients (n=228 genes) to generate HIGH and LOW expression tumor clusters in an independent cohort of 3232 primary colorectal tumors (CMS-3232; [2]) using the k-means algorithm (n=2)) with annotated CMS status and survival. The HIGH and LOW expression subgroups were then analyzed for the distribution of CMS1-4 and time-to-relapse and overall survival (OS). Survival curves were compared using the log-rank test and hazard ratios (HRs) were estimated by Cox regression. The assumption of proportional hazards was assessed according to inspection of Schoenfeld residual plots.

References

1. Ubink I, Elias SG, Moelans CB, *et al.* A Novel Diagnostic Tool for Selecting Patients With Mesenchymal-Type Colon Cancer Reveals Intratumor Subtype Heterogeneity. J Natl Cancer Inst 2017;109(8).

2. Guinney J, Dienstmann R, Wang X, *et al.* The consensus molecular subtypes of colorectal cancer. Nat Med 2015;21(11):1350-6.

3. Subramanian A, Tamayo P, Mootha VK, *et al.* Gene set enrichment analysis: a knowledge-based approach for interpreting genome-wide expression profiles. Proc Natl Acad Sci U S A 2005;102(43):15545-50.

4. Bindea G, Mlecnik B, Tosolini M, *et al.* Spatiotemporal dynamics of intratumoral immune cells reveal the immune landscape in human cancer. Immunity 2013;39(4):782-95.

Supplementary Table S1. In- and exclusion criteria for treatment with Imatinib in patients with CMS4 colon cancer in the ImPACCT trial.

Inclusion criteria	Exclusion criteria
- Male or female aged ≥18 years;	- The presence of synchronous distant metastases;
- Histologically proven adenocarcinoma of the colon;	- Current hospital standard of care dictates that subject should undergo any neoadjuvant therapy;
- Completed cancer staging with CT-abdomen and CT-thorax/X-thorax according to hospital's standard of care;	- Concurrent participation in another clinical trial using any medicinal product, or participation in such a trial in the period of three months prior to the current trial;
- Confirmed eligibility for surgery with curative intent as deemed by the hospital's multidisciplinary board review;	- Women who are pregnant, plan to become pregnant or are lactating during the study or for up to 30 days after the last dose of imatinib;
- Test positive for CMS4 subtype;	- Known HIV or Hepatitis B/C infection;
- ≥4 properly stored pre-treatment biopsies for gene expression analysis/ELISA;	- Known symptomatic congestive heart failure;
- WHO performance status 0 or 1;	- Co-morbidity requiring concomitant treatment with drugs that act as strong inducers of CYP3A4 or with drugs with a narrow therapeutic range influenced by imatinib.
- Adequate haematology status and organ function (defined as: normal creatinine clearance (≥60 ml/min (MDRD)), ALAT within 2.5× upper limit of normal (ULN), PT-INR < 1.5, leukocytes >1,5*10^9/L, Hb > 6.0 mmol/L, platelets >100*10^9/L);	
- Willingness and ability to comply with scheduled visits, treatment plans and laboratory tests;	
- Provision of written informed consent.	

ELISA, enzyme-linked immunosorbent assay; WHO, World Health Organization, MDRD, Modification of Diet in Renal Disease; ALAT, Alanine Transaminase; PT-INR, Prothrombin time International Normalized Ratio; Hb, Hemoglobin.

Supplementary Table S2. Gene set signatures used in the analyses of the ImPACCT study.

Circulating cell cluster	HALLMARK_MYC_TARGETS_V1	HALLMARK_MTORC1 SIGNALING	CMS4 upregulated genes	WNT	KEGG cell cycle	mTOR TOP targets	KEGG adherens junctions	Desmosome	4-gene signature
XBP1	ABCE1	ABCF2	ACTA2	AURKB	ABL1	EEF1A1A	ACP1	DSC1	PDGFRA
ERBB3	ACP1	ACACA	ADAM12	BYSL	ANAPC1	EEF1B2A	ACTB	DSC2	PDGFRB
KRT19	AIMP2	ACLY	ADAMTS12	C12ORF14	ANAPC10	EEF1GA	ACTG1	DSC3	PDGFC
JUP	AP3S1	ACSL3	AEBP1	C16ORF35	ANAPC11	EEF2A	ACTN1	DSG1	KIT
TACSTD2	APEX1	ACTR2	AKAP12	C1ORF33	ANAPC2	EF1DA	ACTN2	DSG2	
SERPINB6	BUB3	ACTR3	ANK2	C20ORF64	ANAPC4	EIF1AY	ACTN3	DSG3	
CHP1	C1QBP	ADD3	ANTXR1	CAD	ANAPC5	EIF2AA	ACTN4	DSG4	
PSME3	CAD	ADIPOR2	AOC3	CD320	ANAPC7	EIF2B3	ACVR1B	DSP	
MLPH	CANX	AK4	AQP1	CD44	ATM	EIF2S2	ACVR1C	JUP	
SSR4	CBX3	ALDOA	ARMCX1	CDC25A	ATR	EIF2S3	BAIAP2	PKP1	
RPS4X	CCNA2	ARPC5L	ASPN	CDCA7	BUB1	EIF3AA	CDC42	PKP2	
RPL32	CCT2	ASNS	BGN	CDK4	BUB1B	EIF3C	CDH1	PKP3	
RGL2	CCT3	ATP2A2	BICC1	CDKN3	BUB3	EIF3D	CREBBP	PKP4	
PSMD4	CCT4	ATP5MC1	BNC2	CDT1	CCNA1	EIF3EA	CSNK2A1		
NUCB2	CCT5	ATP6V1D	BOC	CGI-96	CCNA2	EIF3FA	CSNK2A2		
LRPAP1	CCT7	AURKA	C1R	CIRH1A	CCNB1	EIF3HA	CSNK2B		
UBE2L3	CDC20	BCAT1	C1S	CLDN2	CCNB2	EIF3K	CTNNA1		
HSP90AA1	CDC45	BHLHE40	C3	COG8	CCNB3	EIF3L	CTNNA2		
SDHA	CDK2	BTG2	CACNA2D1	CRTAP	CCND1	EIF4A2	CTNNA3		
TUG1	CDK4	BUB1	CALD1	CTPS	CCND2	EIF4BA	CTNNB1		
MYL6	CLNS1A	CACYBP	CCDC80	DCUN1D5	CCND3	GNB2L1A	CTNND1		
AGR2	CNBP	CALR	CLDN11	DDX10	CCNE1	HNRNPA1A	EGFR		
ELF3	COPS5	CANX	CNN1	DDX20	CCNE2	PABPC1A	EP300		
KRT18	COX5A	CCNF	COL14A1	DDX21	CCNH	PABPC4A	ERBB2		
ATP5A1	CSTF2	CCNG1	COL15A1	DDX56	CDC14A	QARSA	FARP2		

Circulating cell cluster	HALLMARK_MYC_TARGETS_V1	HALLMARK_MTORC1 SIGNALING	CMS4 upregulated genes	WNT	KEGG cell cycle	mTOR TOP targets	KEGG adherens junctions	Desmosome	4-gene signature
RPL24	CTPS1	CCT6A	COL1A1	DKC1	CDC14B	RPL10A	FER		
EIF3F	CUL1	CD9	COL1A2	EIF5A2	CDC16	RPL12A	FGFR1		
C20orf24	CYC1	CDC25A	COL3A1	ENC1	CDC20	RPL13A	FYN		
PAPOLA	DDX18	CDKN1A	COL5A1	ETS2	CDC23	RPL14A	IGF1R		
CHCHD2	DDX21	CFP	COL5A2	ETV4	CDC25A	RPL15A	INSR		
SNAP23	DEK	COPS5	COL6A3	FARSLA	CDC25B	RPL17A	IQGAP1		
BTG2	DHX15	CORO1A	COL8A1	FLJ10774	CDC25C	RPL18	LEF1		
RPS6	DUT	CTH	COL8A2	FLJ20249	CDC26	RPL18A	LMO7		
LRRFIP1	EEF1B2	CTSC	COLEC12	FLJ20315	CDC27	RPL19	MAP3K7		
NHP2L1	EIF1AX	CXCR4	CRISPLD1	FLJ20425	CDC6	RPL21	MAPK1		
C19orf43	EIF2S1	CYB5B	CRYAB	FLJ23476	CDC7	RPL22A	MAPK3		
HSP90AB1	EIF2S2	CYP51A1	CTSK	FOXQ1	CDK2	RPL23A	MET		
ARPC2	EIF3B	DAPP1	CXCL12	GEMIN5	CDK4	RPL24	MLLT4		
RAC1	EIF3D	DDIT3	CYP1B1	HEATR1	CDK6	RPL26	NLK		
NDUFB9	EIF3J	DDIT4	DCLK1	HES6	CDK7	RPL27	PARD3		
RAN	EIF4A1	DDX39A	DCN	HIG2	CDKN1A	RPL27A	PTPN1		
NPLOC4	EIF4E	DHCR24	DDR2	HNRPDL	CDKN1B	RPL29A	PTPN6		
CAPNS1	EIF4G2	DHCR7	DPT	HSPC111	CDKN1C	RPL3	PTPRB		
UBC	EIF4H	DHFR	DPYSL3	IMP4	CDKN2A	RPL30	PTPRF		
RHOA	EPRS1	EBP	ECM2	KIAA0179	CDKN2B	RPL31A	PTPRJ		
DSP	ERH	EDEM1	EDNRA	KIAA0690	CDKN2C	RPL32A	PTPRM		
CDIPT	ETF1	EEF1E1	EFEMP1	KIAA1068	CDKN2D	RPL34A	PVRL1		
BANF1	EXOSC7	EGLN3	FBLN1	KIAA1199	CHEK1	RPL35	PVRL2		
HSPA5	FAM120A	EIF2S2	FBN1	LOC150223	CHEK2	RPL36A	PVRL3		
S100A11	FBL	ELOVL5	FBXO32	LOC285958	CREBBP	RPL37A	PVRL4		
MLEC	G3BP1	ELOVL6	FERMT2	LOC389362	CUL1	RPL39A	RAC1		

Circulating cell cluster	HALLMARK_MYC_TARGETS_V1	HALLMARK_MTORC1 SIGNALING	CMS4 upregulated genes	WNT	KEGG cell cycle	mTOR TOP targets	KEGG adherens junctions	Desmosome	4-gene signature
DYNLL1	GLO1	ENO1	FIBIN	LOC56902	DBF4	RPL40	RAC2		
ELOVL5	GNL3	EPRS1	FLNA	MAC30	E2F1	RPL41A	RAC3		
MRPL43	GOT2	ERO1A	FN1	MET	E2F2	RPL4A	RHOA		
TUBA1A	GSPT1	ETF1	FNDC1	METTL1	E2F3	RPL5A	SMAD2		
HNRNPC	H2AZ1	FADS1	FSTL1	MGC13096	EP300	RPL6A	SMAD3		
SIRT3	HDAC2	FADS2	FXYD6	MGC13170	ESPL1	RPL7	SMAD4		
UFC1	HDDC2	FDXR	GEM	MGC2408	FZR1	RPL7AA	SNAI1		
SNF8	HDGF	FGL2	GLT8D2	MGC2574	GADD45A	RPL8A	SNAI2		
HSPA4	HNRNPA1	FKBP2	GPC6	MGC40397	GADD45B	RPL9	SORBS1		
CNOT8	HNRNPA2B1	G6PD	GREM1	MGC4677	GADD45G	RPLP0A	SRC		
NUP93	HNRNPA3	GAPDH	GUCY1A3	MKI67IP	GSK3B	RPLP1A	SSX2IP		
CYTH2	HNRNPC	GBE1	GUCY1B3	MRPL36	HDAC1	RPLP2A	TCF7		
NDRG1	HNRNPD	GCLC	HDGFRP3	MRPS12	HDAC2	RPS10A	TCF7L1		
TOMM6	HNRNPR	GGA2	HMCN1	MRPS26	MAD1L1	RPS11A	TCF7L2		
PRICKLE4	HNRNPU	GLA	HOPX	MRPS30	MAD2L1	RPS12	TGFBR1		
HBXIP	HPRT1	GLRX	HSD17B6	MTVR1	MAD2L2	RPS13	TGFBR2		
CMPK1	HSP90AB1	GMPS	HSPB8	MYBBP1A	MCM2	RPS14A	TJP1		
OS9	HSPD1	GOT1	HTR2B	MYC	MCM3	RPS15	VCL		
PSMA1	HSPE1	GPI	IGFBP5	NLE1	MCM4	RPS15AA	WAS		
TRIB1	IARS1	GSK3B	INHBA	NOB1P	MCM5	RPS16A	WASF1		
NACA	IFRD1	GSR	ISLR	NOC3L	MCM6	RPS17A	WASF2		
SKP1	ILF2	GTF2H1	KCTD12	NOL1	MCM7	RPS18	WASF3		
ABHD11	IMPDH2	HK2	LBH	NOL5A	MDM2	RPS19A	WASL		
RPLP0	KARS1	HMBS	LHFP	NOLA1	PCNA	RPS20A	YES1		
RPL11	KPNA2	HMGCR	LMOD1	NS	PKMYT1	RPS21			
PPIA	KPNB1	HMGCS1	LOX	NXT1	PLK1	RPS23			

Circulating cell cluster	HALLMARK_MYC_TARGETS_V1	HALLMARK_MTORC1 SIGNALING	CMS4 upregulated genes	WNT	KEGG cell cycle	mTOR TOP targets	KEGG adherens junctions	Desmosome	4-gene signature
RPL13A	LDHA	HPRT1	LUM	ODC1	PRKDC	RPS24			
RPLP1	LSM2	HSP90B1	MAP1B	PDCD11	PTTG1	RPS25			
TMSB10	LSM7	HSPA4	MEIS1	PEO1	PTTG2	RPS26			
P4HB	MAD2L1	HSPA5	MFAP2	PHLDA1	RB1	RPS27			
IRF1	MCM2	HSPA9	MGP	PMSCL1	RBL1	RPS27AA			
DNM1L	MCM4	HSPD1	MIR100HG	POLR1B	RBL2	RPS28			
SCAMP4	MCM5	HSPE1	MMP2	POLR1C	RBX1	RPS29			
BRK1	MCM6	IDH1	MN1	POLR1D	SFN	RPS2A			
RPL7A	MCM7	IDI1	MOXD1	PPAN	SKP1	RPS3			
MACF1	MRPL23	IFI30	MRGPRF	PPIL1	SKP2	RPS30			
PGRMC1	MRPL9	IFRD1	MSRB3	PSF1	SMAD2	RPS3AA			
RPS19	MRPS18B	IGFBP5	MXRA5	RAB9P40	SMAD3	RPS4XA			
EEF1A1	MYC	IMMT	MYL9	RFP	SMAD4	RPS4Y			
HNRNPM	NAP1L1	INSIG1	MYLK	RHEB	SMC1A	RPS5A			
ZFAND5	NCBP1	ITGB2	NAP1L3	RNU3IP2	SMC1B	RPS6A			
EFNA1	NCBP2	LDHA	NDN	RRP41	TFDP1	RPS7			
RPS3A	NDUFAB1	LDLR	NEXN	SCD	TGFB1	RPS8			
RPL19	NHP2	LGMN	PCDH7	SDCCAG16	TGFB2	RPS9A			
ARG2	NME1	LTA4H	PCOLCE	SF3A2	TGFB3	RPSA			
ARRB1	NOLC1	M6PR	PDE1A	SLC19A1	TP53				
SRRM2	NOP16	MAP2K3	PDGFC	SLC29A2	WEE1				
MAPRE1	NOP56	MCM2	PDLIM3	SLC39A10	YWHAB				
IDS	NPM1	MCM4	PEG3	SLC3A2	YWHAE				
STRAP	ODC1	ME1	PHLDB2	SLC7A5	YWHAG				
ACTG1	ORC2	MLLT11	PLN	SORD	YWHAH				
RPSA	PA2G4	MTHFD2	PPP1R3C	SOX4	YWHAQ				

Circulating cell cluster	HALLMARK_MYC_TARGETS_V1	HALLMARK_MTORC1 SIGNALING	CMS4 upregulated genes	WNT	KEGG cell cycle	mTOR TOP targets	KEGG adherens junctions	Desmosome	4-gene signature
TLN1	PABPC1	MTHFD2L	PRELP	SOX9	YWHAZ				
RBM8A	PABPC4	NAMPT	PRICKLE1	SRM					
YIF1B	PCBP1	NFIL3	PTGIS	TEAD4					
RPL3	PCNA	NFKBIB	RAB31	TERE1					
RPS25	PGK1	NFYC	RARRES2	TGIF					
RPL23	PHB	NIBAN1	RBMS1	TM4SF9					
ETF1	PHB2	NMT1	RBMS3	TREX2					
RPL35	POLD2	NUFIP1	RUNX1T1	TRMT1					
IMPDH2	POLE3	NUP205	S1PR3	WDR12					
CD63	PPIA	NUPR1	SDC2	WDR3					
TMED10	PPM1G	P4HA1	SERPINF1	WDR74					
UQCRC2	PRDX3	PDAP1	SERPING1	WDR77					
SSBP1	PRDX4	PDK1	SFRP2	XPOT					
HMGN1	PRPF31	PFKL	SFRP4	YAP1					
SLC1A4	PRPS2	PGK1	SGCE	ZNF259					
NDUFA13	PSMA1	PGM1	SLIT2	ZNF511					
EEF1B2	PSMA2	PHGDH	SNAI2	ZNF593					
ALDOA	PSMA4	PIK3R3	SPARCL1	ZNF703					
PUF60	PSMA6	PITPNB	SPOCK1						
NEDD8	PSMA7	PLK1	SSPN						
NDUFS2	PSMB2	PLOD2	STON1						
CNPY2	PSMB3	PNO1	SULF1						
ATP6V1F	PSMC4	PNP	TAGLN						
ARPP19	PSMC6	POLR3G	TCEAL7						
EZR	PSMD1	PPA1	TGFB1I1						
AZIN1	PSMD14	PPIA	THBS1						

Circulating cell cluster	HALLMARK_MYC_TARGETS_V1	HALLMARK_MTORC1 SIGNALING	CMS4 upregulated genes	WNT	KEGG cell cycle	mTOR TOP targets	KEGG adherens junctions	Desmosome	4-gene signature
WHSC1L1	PSMD3	PPP1R15A	THBS2						
MXD3	PSMD7	PRDX1	THY1						
EIF3J	PSMD8	PSAT1	TIMP2						
GSK3A	PTGES3	PSMA3	TIMP3						
MYL12B	PWP1	PSMA4	TMEM47						
CAND1	RACK1	PSMB5	TNC						
RAB13	RAD23B	PSMC2	TNS1						
SLC35B1	RAN	PSMC4	TPM2						
CS	RANBP1	PSMC6	VCAN						
TPD52	RFC4	PSMD12	WISP1						
ITFG3	RNPS1	PSMD13	WWTR1						
CLDN4	RPL14	PSMD14	ZCCHC24						
AATF	RPL18	PSME3	ZFPM2						
SPTAN1	RPL22	PSMG1	ZNF521						
GAS5	RPL34	PSPH							
TMCO1	RPL6	QDPR							
FLOT1	RPLP0	RAB1A							
PPP2CB	RPS10	RDH11							
GTF3A	RPS2	RIT1							
CIAO1	RPS3	RPA1							
TSPAN1	RPS5	RPN1							
SEPHS2	RPS6	RRM2							
TBCA	RRM1	RRP9							
41897	RRP9	SC5D							
FAF2	RSL1D1	SCD							
COX7A2L	RUVBL2	SDF2L1							

Circulating cell cluster	HALLMARK_MYC_TARGETS_V1	HALLMARK_MTORC1 SIGNALING	CMS4 upregulated genes	WNT	KEGG cell cycle	mTOR TOP targets	KEGG adherens junctions	Desmosome	4-gene signature
GNAI2	SERBP1	SEC11A							
PPM1G	SET	SERP1							
CAST	SF3A1	SERPINH1							
SPATA20	SF3B3	SHMT2							
REEP5	SLC25A3	SKAP2							
NCOA6	SMARCC1	SLA							
ERGIC3	SNRPA	SLC1A4							
TMEM115	SNRPA1	SLC1A5							
ZNF385A	SNRPB2	SLC2A1							
PRRC2C	SNRPD1	SLC2A3							
NAA60	SNRPD2	SLC37A4							
TNFRSF1A	SNRPD3	SLC6A6							
FNBP1	SNRPG	SLC7A11							
OCIAD1	SRM	SLC7A5							
ITGB5	SRPK1	SLC9A3R1							
UBE2D3	SRSF1	SORD							
AP1B1	SRSF2	SQLE							
TRPS1	SRSF3	SQSTM1							
TAF9	SRSF7	SRD5A1							
GPR56	SSB	SSR1							
SERINC1	SSBP1	STARD4							
NRD1	STARD7	STC1							
DCAF5	SYNCRIP	STIP1							
GDI1	TARDBP	SYTL2							
CLU	TCP1	TBK1							
RPL27A	TFDP1	TCEA1							

Circulating cell cluster	HALLMARK_MYC_TARGETS_V1	HALLMARK_MTORC1 SIGNALING	CMS4 upregulated genes	WNT	KEGG cell cycle	mTOR TOP targets	KEGG adherens junctions	Desmosome	4-gene signature
TXN	TOMM70	TES							
RPL7	TRA2B	TFRC							
JUND	TRIM28	TM7SF2							
RPL18	TUFM	TMEM97							
RPL28	TXNL4A	TOMM40							
PSMB2	TYMS	TPI1							
NBPF10	U2AF1	TRIB3							
RPS10	UBA2	TUBA4A							
ERP29	UBE2E1	TUBG1							
RAD21	UBE2L3	TXNRD1							
HNRNPR	USP1	UBE2D3							
RAD9A	VBP1	UCHL5							
CDC37	VDAC1	UFM1							
SF3B2	VDAC3	UNG							
BAG1	XPO1	USO1							
CCDC84	XPOT	VLDLR							
METTL9	XRCC6	WARS1							
PRLR	YWHAE	XBP1							
SON	YWHAQ	YKT6							
CYB5R1									
SNW1									
TCP1									
CDC16									
RPL14									
RPS26									
TTC3									

Circulating cell cluster	HALLMARK_MYC_TARGETS_V1	HALLMARK_MTORC1 SIGNALING	CMS4 upregulated genes	WNT	KEGG cell cycle	mTOR TOP targets	KEGG adherens junctions	Desmosome	4-gene signature
KIAA1324									
C17orf49									
RPS5									
PEBP1									
RPL22									
ATP1B1									
CSNK1A1									
NQO1									
CANX									
SRP14									
DYNLRB1									
UBA1									
41884									
PPP2CA									
TOMM20									
UQCRFS1									
HN1									
FKBP4									
GDI2									
PSMA6									
PSME2									
CHD4									
RER1									
NDUFB2									
HINT1									
SRSF7									

Circulating cell cluster	HALLMARK_MYC_TARGETS_V1	HALLMARK_MTORC1 SIGNALING	CMS4 upregulated genes	WNT	KEGG cell cycle	mTOR TOP targets	KEGG adherens junctions	Desmosome	4-gene signature
SUPT5H									
COX4I1									
APLP2									
SEPW1									
PRELID1									
UNKL									
TMEM50A									
FIS1									
CHMP2A									
CTNNA1									
PYCR1									
SUGT1									
PRDX2									
TRIM28									
PLP2									
WDR34									
PSMD8									
CRIP1									
KCP									
POLR2L									
BHLHE40									
TXNL4A									
ACBD6									
NDUFA1									
CREB3									
NAPA									

Circulating cell cluster	HALLMARK_MYC_TARGETS_V1	HALLMARK_MTORC1 SIGNALING	CMS4 upregulated genes	WNT	KEGG cell cycle	mTOR TOP targets	KEGG adherens junctions	Desmosome	4-gene signature
ATP5J2									
AFTPH									
PGD									
TFG									
SAP30L									
JTB									
ANAPC5									
IDH3B									
MAGT1									
GORASP2									
ANXA2									
LASP1									
DCAF7									
MTIF3									
BICD2									
STX16									
ATP6V1C1									
ARF3									
RNF139									
NAGK									
PNRC2									
DNAJC12									
RAB7A									
TUSC3									
CDK2AP1									
NDEL1									

Circulating cell cluster	HALLMARK_MYC_TARGETS_V1	HALLMARK_MTORC1 SIGNALING	CMS4 upregulated genes	WNT	KEGG cell cycle	mTOR TOP targets	KEGG adherens junctions	Desmosome	4-gene signature
URI1									
LINC00094									
WARS									
FAM102A									
TOR1A									
RHEB									
FAM127A									
ARPC4									
NSD1									
AKIRIN1									
ID2									
ACTN1									
SCRIB									
SAP30BP									
KIAA1244									
PQBP1									
ACTN4									
RAB10									
LRRC37BP1									
RHBDD2									
CDCP1									
ZKSCAN1									
CHPF									
IRX5									
C17orf28									
PRSS53									

Circulating cell cluster	HALLMARK_MYC_TARGETS_V1	HALLMARK_MTORC1 SIGNALING	CMS4 upregulated genes	WNT	KEGG cell cycle	mTOR TOP targets	KEGG adherens junctions	Desmosome	4-gene signature
VPS37C									
VKORC1									
ABHD12									
BAIAP2L1									
LIMS1									
CIR1									
ATP6V1E1									
NUDT9									
SDF2									
RABL5									
GNB2L1									
PIGX									
PTPN2									
BTF3									
SLC25A39									
PSME1									
ZNF274									
SCNM1									
NDUFA4									
CCT3									
C20orf11									
GOT2									
FBXW4									
YIPF6									
HAX1									
SEL1L									

Circulating cell cluster	HALLMARK_MYC_TARGETS_V1	HALLMARK_MTORC1 SIGNALING	CMS4 upregulated genes	WNT	KEGG cell cycle	mTOR TOP targets	KEGG adherens junctions	Desmosome	4-gene signature
MCM3AP									
ARCN1									
EID1									
ORC4									
ZYX									
PSMD13									
BRP44L									
TTC39A									
TRPM4									

Supplementary Table S3. Kinases correlated with CMS4

Kinases significantly (p<e-6) correlated with CMS4 signature genes					
**CIT566**	**R**	**TCGA286**	**R**	**overlap (55 kinases)**	**overlap (22 tyrosine kinases)**
DDR2	0.929	PDGFRB	0.941	AAK1	AXL
AXL	0.86	DDR2	0.932	AKT3	BTK
AKT3	0.835	AKT3	0.919	AXL	DDR2
PDGFRB	0.823	AXL	0.897	BMPR1B	EPHA3
FGFR1	0.816	PKD2	0.877	BMPR2	EPHA7
PKD2	0.808	TIE1	0.873	BTK	FER
NUAK1	0.804	FGFR1	0.867	DAPK1	FES
LATS2	0.745	NUAK1	0.831	DDR2	FGFR1
TIE1	0.737	FLT4	0.827	DYRK3	FGR
EPHA3	0.659	EPHA3	0.791	EPHA3	FLT1
MAP3K3	0.656	MAP3K3	0.79	EPHA7	FYN
ILK	0.63	PDGFRA	0.757	FER	HCK
FYN	0.595	FGR	0.744	FES	ITK
HCK	0.594	ROR2	0.741	FGFR1	JAK1
PDGFRA	0.584	KDR	0.728	FGR	JAK3
ROR2	0.567	LRRK2	0.724	FLT1	KDR
LRRK2	0.558	HCK	0.721	FYN	KIT
KDR	0.534	BTK	0.716	HCK	PDGFRA
TGFBR1	0.526	FES	0.716	HIPK3	PDGFRB
FGR	0.517	LATS2	0.715	ILK	ROR1
FLT1	0.496	CAMK1G	0.71	IRAK3	ROR2
BMPR2	0.485	FLT1	0.705	ITK	TIE1
JAK3	0.477	SPEG	0.653	JAK1	
BTK	0.453	TGFBR1	0.636	JAK3	
SNRK	0.436	BMPR1B	0.63	KDR	

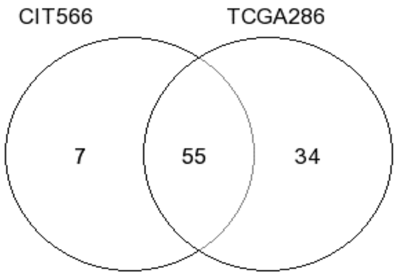

Kinases significantly (p<e-6) correlated with CMS4 signature genes					
**CIT566**	**R**	**TCGA286**	**R**	**overlap (55 kinases)**	**overlap (22 tyrosine kinases)**
ROR1	0.434	BMPR2	0.626	KIT	
PIM1	0.403	WNK3	0.624	LATS2	
FES	0.401	CDKL5	0.608	LRRK2	
OSR1	0.384	ROR1	0.605	MAP3K3	
DAPK1	0.382	ILK	0.604	MAP3K8	
PLK2	0.361	IRAK3	0.602	MARK4	
SPEG	0.351	SNRK	0.602	MAST4	
ULK2	0.347	TTBK2	0.602	NEK1	
TRIO	0.347	PAK3	0.581	NEK7	
IRAK3	0.34	JAK3	0.578	NEK9	
HIPK3	0.336	BRSK1	0.569	NUAK1	
ITK	0.333	FYN	0.569	OSR1	
TTBK2	0.322	CAMK2A	0.567	PDGFRA	
AAK1	0.316	HIPK3	0.551	PDGFRB	
NEK7	0.312	DYRK3	0.532	PIM1	
FER	0.312	ALK	0.527	PKD1	
DYRK3	0.306	MAST4	0.519	PKD2	
MAP3K8	0.302	FLT3	0.512	PLK2	
MAPKAPK2	0.294	EPHA5	0.511	ROCK1	
KIT	0.288	CAMK4	0.508	ROR1	
TGFBR2	0.284	ITK	0.5	ROR2	
EPHA7	0.282	EPHB6	0.491	SNRK	
ZAK	0.279	NEK1	0.484	SPEG	
NEK9	0.274	TRIO	0.475	TGFBR1	
PLK3	0.272	BMX	0.473	TGFBR2	
MARK4	0.264	EPHA7	0.452	TIE1	
PKN1	0.256	ROCK1	0.451	TRIO	

Kinases significantly (p<e-6) correlated with CMS4 signature genes					
**CIT566**	**R**	**TCGA286**	**R**	**overlap (55 kinases)**	**overlap (22 tyrosine kinases)**
BMPR1B	0.255	EPHA6	0.447	TTBK2	
PINK1	0.25	GCK	0.447	ULK1	
MAST4	0.244	KIT	0.44	ULK2	
ULK1	0.242	PHKG1	0.432		
ROCK1	0.236	PKD1	0.427		
NEK1	0.231	LATS1	0.42		
RNASEL	0.224	TGFBR2	0.414		
JAK1	0.22	HIPK4	0.412		
MAP3K6	0.218	OSR1	0.404		
PKD1	0.216	MUSK	0.402		
		FER	0.399		
		MAP3K2	0.399		
		NEK7	0.398		
		RET	0.387		
		DAPK1	0.386		
		JAK1	0.381		
		PIM1	0.375		
		IGF1R	0.371		
		DAPK3	0.369		
		NEK9	0.367		
		BLK	0.363		
		ATM	0.361		
		AAK1	0.356		
		WNK1	0.355		
		NRBP1	0.35		
		ULK1	0.349		
		PLK2	0.348		

Kinases significantly (p<e-6) correlated with CMS4 signature genes					
CIT566	R	TCGA286	R	overlap (55 kinases)	overlap (22 tyrosine kinases)
		ZAP70	0.348		
		EPHA4	0.334		
		PIM2	0.325		
		MAP3K1	0.313		
		MYO3A	0.311		
		TAF1L	0.308		
		ULK2	0.305		
		MARK4	0.304		
		PIK3R4	0.301		
		MAP3K8	0.3		

Supplementary Table S4. Kd values of 72 tyrosine kinase inhibitors for CMS4 related kinases.

	# targets		selectivity score	KIT	PDGFRA	PDGFRB	FLT1	DDR2	KDR	AXL	TIE1	FGR	FYN	HCK	BTK	EPHA3	EPHA7	FER	FES	FGFR1	ITK	JAK1	JAK3
	all	<300	σ (3 uM)																				
VX-745	4	0	0.0233		8800	8400						1300	2100										
PTK-787	5	5	0.0311	5.1	96	25	9.6		62														
MLN-518	6	4	0.057	2.7	2.4	4.5	3100	120		6300													
**Imatinib***	6	4	0.057	13	31	14		15				2400	3100										
AB-1010	8	5	0.0622	8.1	25	8.4		26				640	140	690		2500							
CP-690550	3	2	0.0622										1100									1.6	0.16
AC220	9	5	0.0751	4.8	11	7.7	41	1100	87		3200					6500	3700						
AMG-706	10	5	0.0777	3.7	10	9.1	12		26			6900	2800	8600						6200		5200	
BMS-540215	9	6	0.0855	36	11	50	10		5		1100			2700			1700			99			
MLN-8054	11	1	0.0984				1000			440	1600	220	1300	1500	4200	2100	1000	4400		2400			
Gefitinib	4	0	0.1114							1800		2600		4400		5500							
AZD-1152HQPA	10	4	0.114	17	38	41	110	6600	500	390	350			3900	1200								
CHIR-265/RAF-265	11	3	0.1166	200	1100	240	800	960	1300		150		2100	1200				8600		4700			
**Nilotinib***	9	5	0.1244	29	180	73		33			1000	320	1600	390		110							
LY-317615	4	0	0.1321			3300				3300										4000			1700
Ki-20227	11	7	0.1347	0.69	0.49	0.29	130	88	18	140		1800	7100	440	7400								
BIRB-796	10	3	0.1373	170	1200	1100	410	33	3900		8.3					880	860			4300			
GSK-1838705A	4	2	0.1373							300								9.3	52				4700
PLX-4720	11	4	0.1399	180	190	200	1900	4100	2100			62	2300	2700					3600	5300			
CI-1033	13	0	0.1606	7800	5200	7500	7500			5700	2200	2800	5300	4200	1600	2100					5600		630
Cediranib	15	8	0.1632	0.38	0.41	0.32	0.74	48	1.1	490	290	1100	1200	590	4300	3700	620			53			
**Sorafenib***	15	7	0.1684	28	62	37	31	6.6	59	4500	68	7800	8400	8500		1900	5300			2800			7300
HKI-272	8	2	0.1813							190	390	1900	6400	490	160			510	590				2000
Erlotinib	12	0	0.1813	1700	1800	1400	4400		5700	4000	850	1100		1800		2400	1400						700
ABT-869	10	7	0.1839	2	4.2	1.9	7.5	3800	8.1	340	110					3500	110						

AG-013736	12	6	0.1969	3.2	0.51	0.57	5.8	5300	5.9	420	97	1800								380	1200		3100
**Pazopanib***	14	6	0.215	2.8	4.9	2	14	98	14		700	1600	2700	5700				2700	1400	990			6900
**Vandetanib***	15	6	0.2358	260	230	88	260	320	820	250	1500	270	360	360	1700	2000	2400			560			
INCB18424	3	2	0.2565													6100						3.4	2
Flavopiridol	4	0	0.2617		5900					3500						3300							1600
**Dasatinib***	13	9	0.2668	0.81	0.47	0.63	5000	3.2	2900			0.5	0.79	0.35	1.4	0.093				3700			640
PHA-665752	8	1	0.3031	3200	2000	1900		7600		110	1200	1900											470
Crizotinib	13	4	0.3212				2300			7.8	110	670	1300		7800	700	470	270	450		2000	330	200
PD-173955	17	11	0.3446	1.8	5.6	1.4	23	12	690		1200	2.4	4.9	3.3	220	7.6		3500	680	62		330	510
VX-680/MK-0457	19	5	0.3472	240	1600	310	100	230	2000	210	270	790	530	6200	4400	1500		4200	7400	550	350	5900	630
CHIR-258/TKI-258	14	7	0.3601	7.5	54	3.8	69	8600	68	3800	4000	190	440	3300						150	530		410
JNJ-28312141	19	11	0.3886	3.6	27	28	53	100	460	5.3		220	340	3700	77	1600	6700	120	1100	3500	490	200	18
SKI-606	15	8	0.4249	420	5100	200		140		52	2900	6.3	11	3.4	4.8	5.8		360	330		1700		1200
PKC-412	14	3	0.4301	220	380	110	450		3200	620	1400	730	2100	720				1200		1600		670	12
PP-242	15	5	0.4352	360	220	78	1200	6100	1200	1200		160	300	190	3200	390				570		300	86
**Foretinib***	18	17	0.4404	2.5	4.5	0.96	3.8	3.6	12	0.093	0.79	40	88	15	76	1	2.5	37	110	690	69		
AST-487	19	12	0.4922	5.4	27	8.1	86	11	200	570	0.29	190	50	880		80	1100	590	360	620	990	74	260
BIBF-1120 (derivative)	19		0.5181	5.7	16	15	63	42	2.9	12	2200	300	630	5300	310	8600		73	1200	92	210	2500	8.2
TG-101348	20		0.5389	130	2700	45	4300	950	5200	160	700	82	38	2100	850	7100	6500	1100	530	280	490	18	74
SU-14813	19		0.5415	0.68	1.1	0.29	4.7	9900	2.3	84	5700	390	2600	2200	4000	2300	1500	34		1900	190	5900	580
Sunitinib	20		0.5959	0.37	0.79	0.075	1.8	2900	1.5	9	3900	270	520	880	2100	2100	2400	1100	960	520	13	6000	1200
R406	20		0.6088	6.8	60	3.3	16	43	40	82	76	33	28	150	190	360	39	130	200	44	400	21	36
KW-2449	20		0.658	53	130	30	73	2100	120	180	300	440	660	4300	1000	810	390	1100	750	370	240	1200	39
TAE-684	20		0.671	110	840	150	550	4100	940	12	15	120	1400	410	23	4000	200	1.4	4.8	47	34	410	17
CEP-701	20		0.8031	150	350	29	120	400	220	35	680	52	84	270	66	160	260	28	370	310	290	8.8	2.3
Staurosporine	20		0.8782	19	10	1.8	150	42	220	6.8	65	17	33	20	210	27	630	24	23	90	19	6.1	12
GSK-461364A	2		0.101			2900					1400												
BIBW-2992	2		0.0751							5300				2200									
CI-1040	1		0.0078			3100																	
GSK-690693	1		0.1451			3900																	

Unspecific (σ>0.5)

SB-203580	1	0.0933		5000			Ineffective (<3 targets and all IC50 >1000)
BI-2536	1	0.1425			1900		
BMS-387032/SNS-032	1	0.1606	9900				
LY-333531	1	0.1969				1200	
A-674563	1	0.2772				1500	
AZD-6244/ARRY-886	0	0.0052					
GW-2580	0	0.0104					
SGX-523	0	0.0104					
Lapatinib	0	0.0155					
MLN-120B	0	0.0207					
GDC-0879	0	0.0415					
BMS-345541	0	0.044					
PI-103	0	0.057					
GDC-0941	0	0.0803					
AT-7519	0	0.0933					
R547	0	0.1192					
TG-100-115	0	0.1321					

Supplementary Table S5. FDA approved tyrosine kinase inhibitors against CMS4 related kinases.

	# targets		selectivity score	Kd for CMS4 tyrosine kinases																			
	all	<300	σ (3 uM)	KIT	PDGFRA	PDGFRB	FLT1	DDR2	KDR	AXL	TIE1	FGR	FYN	HCK	BTK	EPHA3	EPHA7	FER	FES	FGFR1	ITK	JAK1	JAK3
**Dasatinib**	13	9	0.2668	0.81	0.47	0.63	5000	3.2	2900			0.5	0.79	0.35	1.4	0.093				3700			640
**Vandetanib**	15	6	0.2358	260	230	88	260	320	820	250	1500	270	360	360	1700	2000	2400			560			
**Pazopanib**	14	6	0.215	2.8	4.9	2	14	98	14		700	1600	2700	5700				2700	1400	990			6900
**Sorafenib**	15	7	0.1684	28	62	37	31	6.6	59	4500	68	7800	8400	8500		1900	5300			2800			7300
**Nilotinib**	9	5	0.1244	29	180	73		33			1000	320	1600	390		110							
**Imatinib**	6	4	0.057	13	31	14		15				2400	3100										

Supplementary Table S6. Patient characteristics of all patients who underwent additional research biopsies for a suspect lesion during colonoscopy in the ImPACCT study.

**Characteristic**	N	N = 70[1]
Age at diagnosis, median (min-max)	69	70 (48-86)
Sex, n (%)	69	
Male		39 (57)
Female		30 (43)
Diagnosis, n (%)	70	
Colorectal Cancer		64 (91)
Adenoma		3 (4.3)
Neuroendocrine tumor		3 (4.3)
Sidedness, n (%)	70	
Right colon		36 (51)
Left colon		29 (41)
Rectum		5 (7.1)
(Serious) Adverse Event after colonoscopy, median (min-max)	69	2 (2.9)

[1]Median (Minimum-Maximum); [2] n (%)

Table S7. Differentially expressed genes in PRE- vs POST-imatinib biopsies[a]

Ensembl ID	hugo	corrected-pvalue	foldchange	sublist	Ensembl ID	hugo	corrected-pvalue	foldchange	sublist
ENSG00000236976	RP11-165H4,2	0.000254561	-9.604872494	POST>PRE	ENSG00000199161	MIR126	1.74E-05	43.55143247	PRE>POST
ENSG00000199568	RNU5A-1	0.000554939	-4.983270179	POST>PRE	ENSG00000270706	CTD-2301A4,5	7.06E-06	14.92752009	PRE>POST
ENSG00000224993	RPL29P12	0.000278115	-4.306487765	POST>PRE	ENSG00000271687	MTND5P10	6.01E-06	14.86090339	PRE>POST
ENSG00000223001	RNU2-61P	0.000169927	-1.846289845	POST>PRE	ENSG00000207757	MIR93	0.000443905	13.85513958	PRE>POST
ENSG00000230364	RPL4P3	0.000692985	-1.626980673	POST>PRE	ENSG00000260035	CTD-2651B20,6	0.000371415	13.45459821	PRE>POST
ENSG00000222414	RNU2-59P	7.31E-05	-1.533473725	POST>PRE	ENSG00000258565	BLZF2P	0.000412976	9.810880073	PRE>POST
ENSG00000202538	RNU4-2	0.000137045	-1.487956627	POST>PRE	ENSG00000207870	MIR221	9.60E-05	8.503229711	PRE>POST
ENSG00000228502	EEF1A1P11	0.000356147	-1.187913785	POST>PRE	ENSG00000213600	U73169,1	0.0001527	7.992931264	PRE>POST
ENSG00000250182	EEF1A1P13	0.000132623	-1.16049888	POST>PRE	ENSG00000199133	MIRLET7D	7.59E-09	7.854577158	PRE>POST
ENSG00000172062	SMN1	5.20E-05	-1.151810902	POST>PRE	ENSG00000213538	KRT8P41	0.000924539	7.51902616	PRE>POST
ENSG00000086300	SNX10	0.000354664	-1.150187298	POST>PRE	ENSG00000223916	RP11-353H3,1	0.000508765	7.256642747	PRE>POST
ENSG00000200434	RNA5-8SP2	0.000439784	-1.146066804	POST>PRE	ENSG00000270188	MTRNR2L11	3.21E-05	6.612767625	PRE>POST
ENSG00000099194	SCD	0.000269303	-1.138903323	POST>PRE	ENSG00000259045	RP11-320M16,1	2.76E-05	5.599620472	PRE>POST
ENSG00000132763	MMACHC	0.000242187	-1.11763497	POST>PRE	ENSG00000249271	HNRNPA1P44	0.000929322	5.304927967	PRE>POST
ENSG00000127337	YEATS4	0.000888737	-1.112575205	POST>PRE	ENSG00000213414	CTB-47B8,5	0.000233413	5.131195902	PRE>POST
ENSG00000077152	UBE2T	0.00045149	-1.102631672	POST>PRE	ENSG00000207949	MIR214	1.32E-06	4.825396357	PRE>POST
ENSG00000172893	DHCR7	0.000482777	-1.097835013	POST>PRE	ENSG00000210107	MT-TQ	5.41E-09	4.64489647	PRE>POST
ENSG00000167536	DHRS13	0.000216121	-1.091332053	POST>PRE	ENSG00000202566	MIR421	4.86E-05	4.567437877	PRE>POST
ENSG00000124767	GLO1	0.000174367	-1.091067054	POST>PRE	ENSG00000149506	ZP1	9.55E-05	4.409360991	PRE>POST
ENSG00000113272	THG1L	1.79E-06	-1.090617503	POST>PRE	ENSG00000232680	AC002511,3	0.00038122	4.344827134	PRE>POST
ENSG00000224578	HNRNPA1P48	0.000679836	-1.085976338	POST>PRE	ENSG00000264705	RP11-674P19,2	0.000555061	4.280156457	PRE>POST
ENSG00000181163	NPM1	0.00011765	-1.085807739	POST>PRE	ENSG00000109047	RCVRN	2.23E-06	4.184898803	PRE>POST
ENSG00000104408	EIF3E	0.000104648	-1.084494898	POST>PRE	ENSG00000227621	PHBP11	0.000136974	4.168640871	PRE>POST
ENSG00000177888	ZBTB41	0.000181653	-1.08410595	POST>PRE	ENSG00000241853	RP11-147K6,1	2.96E-05	4.061628423	PRE>POST
ENSG00000183337	BCOR	0.000564348	-1.082308197	POST>PRE	ENSG00000210127	MT-TA	3.47E-06	3.959581365	PRE>POST
ENSG00000120942	UBIAD1	0.000483017	-1.082049819	POST>PRE	ENSG00000055955	ITIH4	0.000567161	3.741157775	PRE>POST
ENSG00000099797	TECR	2.12E-05	-1.081929995	POST>PRE	ENSG00000258907	RP11-644F5,15	3.70E-05	3.720864474	PRE>POST

ENSG00000140718	FTO	0.000998131	-1.081492014	POST>PRE	ENSG00000232177	MTND4P24	0.000710434	3.657013536	PRE>POST
ENSG00000107833	NPM3	0.000930045	-1.081416031	POST>PRE	ENSG00000210196	MT-TP	4.40E-10	3.647856139	PRE>POST
ENSG00000152558	TMEM123	8.49E-05	-1.081183477	POST>PRE	ENSG00000236095	RP11-49O14,2	0.000115306	3.638028607	PRE>POST
ENSG00000143799	PARP1	5.36E-05	-1.08084852	POST>PRE	ENSG00000268707	RP11-247A12,7	0.000785554	3.421785321	PRE>POST
ENSG00000185420	SMYD3	0.000826284	-1.079281835	POST>PRE	ENSG00000212769	HMGN2P8	0.00019074	3.189002736	PRE>POST
ENSG00000070756	PABPC1	8.76E-05	-1.078729548	POST>PRE	ENSG00000253144	RP11-281O15,7	1.60E-07	3.143606227	PRE>POST
ENSG00000145912	NHP2	0.00015976	-1.076521113	POST>PRE	ENSG00000223403	MEG9	1.90E-06	3.108359227	PRE>POST
ENSG00000096401	CDC5L	0.000424764	-1.076440602	POST>PRE	ENSG00000077616	NAALAD2	0.000860694	3.071563244	PRE>POST
ENSG00000172239	PAIP1	0.000440391	-1.074742949	POST>PRE	ENSG00000210194	MT-TE	8.00E-06	2.990464658	PRE>POST
ENSG00000091009	RBM27	0.000195369	-1.074230697	POST>PRE	ENSG00000139155	SLCO1C1	0.00038237	2.964054723	PRE>POST
ENSG00000174891	RSRC1	0.000350826	-1.073689331	POST>PRE	ENSG00000260417	CTD-2542L18,1	5.89E-05	2.871631635	PRE>POST
ENSG00000105220	GPI	2.07E-05	-1.071941555	POST>PRE	ENSG00000227973	PIN4P1	0.000108923	2.867925406	PRE>POST
ENSG00000147905	ZCCHC7	0.000510233	-1.071924716	POST>PRE	ENSG00000178404	DDC8	0.00053328	2.842691232	PRE>POST
ENSG00000204392	LSM2	0.000289548	-1.07188329	POST>PRE	ENSG00000265413	RP11-789C17,1	0.000329178	2.83508489	PRE>POST
ENSG00000173726	TOMM20	0.000166941	-1.071066138	POST>PRE	ENSG00000171101	SIGLEC17P	4.21E-05	2.743912026	PRE>POST
ENSG00000173113	TRMT112	0.000286349	-1.070884225	POST>PRE	ENSG00000272219	CTB-181H17,1	0.00024185	2.731198305	PRE>POST
ENSG00000105281	SLC1A5	0.000324238	-1.07067478	POST>PRE	ENSG00000228212	OFD1P17	4.36E-05	2.719485953	PRE>POST
ENSG00000116857	TMEM9	0.000229027	-1.070449158	POST>PRE	ENSG00000207607	MIR200A	3.56E-05	2.671588504	PRE>POST
ENSG00000168061	SAC3D1	0.00096314	-1.069742022	POST>PRE	ENSG00000272568	CTB-113D17,1	0.000617115	2.661181719	PRE>POST
ENSG00000108270	AATF	4.75E-05	-1.069693236	POST>PRE	ENSG00000248734	CTD-2260A17,1	0.000110137	2.640447076	PRE>POST
ENSG00000155906	RMND1	5.57E-05	-1.069533695	POST>PRE	ENSG00000228972	RP1-86D1,2	0.000723807	2.57777234	PRE>POST
ENSG00000137161	CNPY3	0.000695695	-1.069359077	POST>PRE	ENSG00000207730	MIR200B	1.65E-05	2.561446933	PRE>POST
ENSG00000011260	UTP18	0.000311236	-1.069019273	POST>PRE	ENSG00000206147	RP11-106A1,2	1.23E-11	2.462924448	PRE>POST
ENSG00000136628	EPRS	6.03E-05	-1.068941741	POST>PRE	ENSG00000272789	RP11-286H15,1	2.08E-05	2.45983084	PRE>POST
ENSG00000137154	RPS6	6.31E-05	-1.068934966	POST>PRE	ENSG00000233158	RPS24P6	0.000740672	2.459333096	PRE>POST
ENSG00000138430	OLA1	0.00047437	-1.068713426	POST>PRE	ENSG00000255508	MIR3654	5.79E-05	2.420467594	PRE>POST
ENSG00000147677	EIF3H	0.00015984	-1.068710425	POST>PRE	ENSG00000272217	XXBAC-BPG157A10,21	0.000118313	2.394999185	PRE>POST
ENSG00000040341	STAU2	0.000745907	-1.068672737	POST>PRE	ENSG00000242600	MBL1P	0.000566349	2.367637159	PRE>POST
ENSG00000231500	RPS18	4.83E-05	-1.068545954	POST>PRE	ENSG00000257178	MIR10A	0.000260545	2.360942094	PRE>POST
ENSG00000112667	DNPH1	4.22E-05	-1.068000943	POST>PRE	ENSG00000210151	MT-TS1	2.96E-05	2.350121452	PRE>POST

ENSG00000185721	DRG1	0.000207553	-1.067065341	POST>PRE	ENSG00000260144	RP11-361M10,3	0.000739186	2.329176393	PRE>POST
ENSG00000165832	TRUB1	0.000313302	-1.066543442	POST>PRE	ENSG00000255176	AP002954,3	2.96E-05	2.283028108	PRE>POST
ENSG00000152240	HAUS1	0.000398183	-1.066515368	POST>PRE	ENSG00000240823	RN7SL23P	0.000439841	2.267380696	PRE>POST
ENSG00000112996	MRPS30	0.000311057	-1.066421099	POST>PRE	ENSG00000231531	HINT1P1	0.000997876	2.248014902	PRE>POST
ENSG00000167005	NUDT21	0.000760641	-1.066359814	POST>PRE	ENSG00000224628	RP5-854E16,2	4.28E-06	2.227807394	PRE>POST
ENSG00000074201	CLNS1A	0.000110766	-1.066057404	POST>PRE	ENSG00000128262	POM121L9P	0.000274749	2.225874109	PRE>POST
ENSG00000124733	MEA1	0.000592617	-1.065412248	POST>PRE	ENSG00000207648	MIR192	0.000116818	2.220353797	PRE>POST
ENSG00000181610	MRPS23	0.000719028	-1.065097599	POST>PRE	ENSG00000253864	AC131025,8	8.77E-05	2.21862394	PRE>POST
ENSG00000039123	SKIV2L2	0.000483551	-1.064973928	POST>PRE	ENSG00000204882	GPR20	4.59E-05	2.199623428	PRE>POST
ENSG00000150768	DLAT	0.000134634	-1.06459026	POST>PRE	ENSG00000248923	MTND5P11	5.80E-05	2.184432327	PRE>POST
ENSG00000134371	CDC73	3.07E-06	-1.064550488	POST>PRE	ENSG00000255581	RP11-69M1,4	0.000131945	2.178848716	PRE>POST
ENSG00000143486	EIF2D	0.000112168	-1.063913917	POST>PRE	ENSG00000205147	AC016586,1	0.000121205	2.175206804	PRE>POST
ENSG00000076248	UNG	0.000445411	-1.063860981	POST>PRE	ENSG00000265500	SENP3-EIF4A1	0.000727204	2.157631644	PRE>POST
ENSG00000131462	TUBG1	0.000540218	-1.063262336	POST>PRE	ENSG00000271204	RP11-138A9,1	0.00016281	2.151775097	PRE>POST
ENSG00000134910	STT3A	7.67E-06	-1.062933059	POST>PRE	ENSG00000225591	RP11-360D2,2	6.63E-06	2.145596626	PRE>POST
ENSG00000156482	RPL30	5.74E-05	-1.062925105	POST>PRE	ENSG00000235225	AC016712,2	0.000408945	2.123972888	PRE>POST
ENSG00000170584	NUDCD2	0.000319183	-1.062421277	POST>PRE	ENSG00000267280	RP11-332H18,4	0.000259379	2.123866909	PRE>POST
ENSG00000174100	MRPL45	0.000795662	-1.061868317	POST>PRE	ENSG00000270906	RP11-475J5,6	0.000758728	2.123316517	PRE>POST
ENSG00000164944	KIAA1429	0.000866576	-1.061726092	POST>PRE	ENSG00000216657	RP3-406P24,1	0.000683054	2.111930607	PRE>POST
ENSG00000063046	EIF4B	0.000211692	-1.061571321	POST>PRE	ENSG00000243498	UBA52P5	7.86E-05	2.096456523	PRE>POST
ENSG00000113328	CCNG1	0.000629574	-1.061568263	POST>PRE	ENSG00000163239	TDRD10	5.60E-05	2.086139794	PRE>POST
ENSG00000099995	SF3A1	5.57E-05	-1.061520995	POST>PRE	ENSG00000271344	RP11-274B21,9	0.000176832	2.086009726	PRE>POST
ENSG00000086827	ZW10	0.000324568	-1.061490923	POST>PRE	ENSG00000228389	AC068039,4	0.000632839	2.04200617	PRE>POST
ENSG00000149273	RPS3	0.000131484	-1.061149099	POST>PRE	ENSG00000188765	TMSB4XP2	6.04E-07	2.039407102	PRE>POST
ENSG00000149308	NPAT	3.22E-05	-1.061012558	POST>PRE	ENSG00000214140	PRCD	4.28E-05	2.018601179	PRE>POST
ENSG00000127022	CANX	2.97E-05	-1.060594979	POST>PRE	ENSG00000197816	CCDC180	0.00052353	1.998354823	PRE>POST
ENSG00000118482	PHF3	0.000163202	-1.06028071	POST>PRE	ENSG00000178187	ZNF454	0.00045719	1.989161897	PRE>POST
ENSG00000147604	RPL7	0.000185355	-1.059591306	POST>PRE	ENSG00000271778	RP11-379F4,8	0.000323821	1.973101736	PRE>POST
ENSG00000108406	DHX40	0.000513984	-1.059357134	POST>PRE	ENSG00000179571	NBPF17P	0.0005437	1.96245713	PRE>POST
ENSG00000174547	MRPL11	6.91E-05	-1.059177766	POST>PRE	ENSG00000213493	ACTN4P1	1.32E-06	1.951989661	PRE>POST

ENSG00000143751	SDE2	0.00011489	-1.05912558	POST>PRE	ENSG00000210195	MT-TT	6.26E-06	1.948886974	PRE>POST
ENSG00000105676	ARMC6	0.000483824	-1.058861409	POST>PRE	ENSG00000234925	RP11-254B13,3	0.000730854	1.948733341	PRE>POST
ENSG00000140988	RPS2	0.000520415	-1.058849544	POST>PRE	ENSG00000262652	RP13-638C3,2	0.000163106	1.946988225	PRE>POST
ENSG00000143252	SDHC	0.000633885	-1.058808371	POST>PRE	ENSG00000268034	AC005795,1	0.000110367	1.933481488	PRE>POST
ENSG00000156508	EEF1A1	0.000158956	-1.058688527	POST>PRE	ENSG00000213026	CFL1P4	0.000418909	1.925960907	PRE>POST
ENSG00000161016	RPL8	6.33E-05	-1.058621403	POST>PRE	ENSG00000269929	RP11-2B6,2	3.76E-10	1.916691063	PRE>POST
ENSG00000067704	IARS2	0.000267209	-1.058538594	POST>PRE	ENSG00000237493	RP11-603J24,7	9.24E-05	1.916523364	PRE>POST
ENSG00000108107	RPL28	0.000866714	-1.058350071	POST>PRE	ENSG00000165646	SLC18A2	0.000350745	1.910069392	PRE>POST
ENSG00000198242	RPL23A	1.87E-05	-1.058034932	POST>PRE	ENSG00000234036	TXNP6	3.65E-06	1.892679138	PRE>POST
ENSG00000056097	ZFR	0.000197531	-1.057839361	POST>PRE	ENSG00000205853	RFPL3S	0.000806472	1.871651347	PRE>POST
ENSG00000184508	HDDC3	0.000747314	-1.057172684	POST>PRE	ENSG00000262902	RP11-750B16,1	8.46E-06	1.871282646	PRE>POST
ENSG00000167815	PRDX2	0.000464291	-1.057109995	POST>PRE	ENSG00000224856	RP4-796I17,5	0.000188195	1.860559867	PRE>POST
ENSG00000118181	RPS25	3.19E-06	-1.057105056	POST>PRE	ENSG00000073146	MOV10L1	0.000727413	1.85833356	PRE>POST
ENSG00000110107	PRPF19	0.000926765	-1.056982352	POST>PRE	ENSG00000154263	ABCA10	0.000403134	1.851809282	PRE>POST
ENSG00000175334	BANF1	0.000558524	-1.056778195	POST>PRE	ENSG00000253683	CTB-79E8,3	3.15E-05	1.844859898	PRE>POST
ENSG00000168275	COA6	0.000367948	-1.056209583	POST>PRE	ENSG00000230148	HOXB-AS1	0.000427665	1.843219622	PRE>POST
ENSG00000149136	SSRP1	0.000963306	-1.056189372	POST>PRE	ENSG00000185839	RP4-592A1,2	5.76E-05	1.839708344	PRE>POST
ENSG00000204628	GNB2L1	2.79E-05	-1.055852171	POST>PRE	ENSG00000224004	ATP5C1P1	0.000786488	1.823811779	PRE>POST
ENSG00000136504	KAT7	0.000258722	-1.055801936	POST>PRE	ENSG00000231841	RP11-206F17,2	0.000814837	1.820735583	PRE>POST
ENSG00000121481	RNF2	0.000397635	-1.055798181	POST>PRE	ENSG00000241511	RPS15AP24	1.88E-05	1.818989425	PRE>POST
ENSG00000163125	RPRD2	0.00059038	-1.05546742	POST>PRE	ENSG00000215895	RP11-334L9,1	2.39E-05	1.811444551	PRE>POST
ENSG00000198876	DCAF12	5.91E-05	-1.055237373	POST>PRE	ENSG00000229097	CALM2P2	2.24E-05	1.801498733	PRE>POST
ENSG00000084463	WBP11	0.000396584	-1.055014021	POST>PRE	ENSG00000272148	RP11-195B17,1	0.000413117	1.801138769	PRE>POST
ENSG00000198755	RPL10A	2.33E-05	-1.054862181	POST>PRE	ENSG00000230630	DNM3OS	2.00E-05	1.787618515	PRE>POST
ENSG00000262814	MRPL12	0.000633178	-1.054824562	POST>PRE	ENSG00000214019	RP6-218J18,2	3.37E-05	1.777792536	PRE>POST
ENSG00000113407	TARS	0.000164785	-1.054628716	POST>PRE	ENSG00000215840	RP11-122G18,7	0.00012098	1.776833043	PRE>POST
ENSG00000082515	MRPL22	0.000188459	-1.054356044	POST>PRE	ENSG00000258427	RBM8B	1.90E-06	1.757375195	PRE>POST
ENSG00000164587	RPS14	0.000134082	-1.054140751	POST>PRE	ENSG00000262951	RP11-670E13,2	0.000202437	1.755646673	PRE>POST
ENSG00000065427	KARS	0.000645438	-1.053780614	POST>PRE	ENSG00000091844	RGS17	0.000518984	1.74953042	PRE>POST
ENSG00000069329	VPS35	0.00026948	-1.053586692	POST>PRE	ENSG00000241255	RP1-89D4,1	1.01E-05	1.747567122	PRE>POST

ENSG00000133318	RTN3	0.000247025	-1.053487575	POST>PRE	ENSG00000224781	EIF4A2P4	0.000483199	1.747494177	PRE>POST
ENSG00000168003	SLC3A2	0.000727132	-1.053393842	POST>PRE	ENSG00000239246	RP11-464D20,2	9.91E-06	1.730861132	PRE>POST
ENSG00000181472	ZBTB2	0.000373807	-1.053299472	POST>PRE	ENSG00000105988	NHP2P1	8.54E-05	1.729274433	PRE>POST
ENSG00000107625	DDX50	0.000484222	-1.053040796	POST>PRE	ENSG00000226318	RP11-474D14,2	0.000674892	1.724170917	PRE>POST
ENSG00000135486	HNRNPA1	0.000667245	-1.052970067	POST>PRE	ENSG00000253284	RP11-282K24,3	0.000634293	1.722366085	PRE>POST
ENSG00000118363	SPCS2	0.00016568	-1.052857467	POST>PRE	ENSG00000247157	RP11-434C1,1	0.000544152	1.71579926	PRE>POST
ENSG00000107341	UBE2R2	3.02E-05	-1.052806008	POST>PRE	ENSG00000169253	RP11-220D10,1	6.27E-06	1.712696184	PRE>POST
ENSG00000113712	CSNK1A1	0.000292449	-1.052770904	POST>PRE	ENSG00000272512	RP11-54O7,17	0.000929075	1.711780936	PRE>POST
ENSG00000150753	CCT5	0.000264804	-1.052647689	POST>PRE	ENSG00000244192	RP11-114H7,1	1.75E-07	1.710606311	PRE>POST
ENSG00000089220	PEBP1	0.000533284	-1.052577531	POST>PRE	ENSG00000231313	CLIC1P1	0.000110167	1.702412812	PRE>POST
ENSG00000132912	DCTN4	0.000275059	-1.052318621	POST>PRE	ENSG00000135502	SLC26A10	0.000172816	1.698808136	PRE>POST
ENSG00000110713	NUP98	0.000431488	-1.052154198	POST>PRE	ENSG00000267281	RP11-793H13,10	0.000458937	1.698659266	PRE>POST
ENSG00000115268	RPS15	0.000368565	-1.052061844	POST>PRE	ENSG00000272871	RP11-408A13,4	4.64E-08	1.684643006	PRE>POST
ENSG00000130175	PRKCSH	3.33E-06	-1.051905189	POST>PRE	ENSG00000255967	RP11-438L7,3	0.00011433	1.683808521	PRE>POST
ENSG00000135108	FBXO21	0.000444731	-1.051876401	POST>PRE	ENSG00000198976	MIR429	0.000148846	1.671947489	PRE>POST
ENSG00000112306	RPS12	0.000382883	-1.051794668	POST>PRE	ENSG00000257354	RP11-631N16,2	0.000611028	1.667237905	PRE>POST
ENSG00000167526	RPL13	7.06E-05	-1.051427585	POST>PRE	ENSG00000236814	RP11-446E9,1	0.000563533	1.663736583	PRE>POST
ENSG00000149806	FAU	5.95E-08	-1.051289311	POST>PRE	ENSG00000271862	RP11-343L5,2	0.000314921	1.648508312	PRE>POST
ENSG00000116251	RPL22	5.62E-05	-1.051076202	POST>PRE	ENSG00000249669	MIR143HG	2.37E-05	1.647448503	PRE>POST
ENSG00000112339	HBS1L	0.00037232	-1.051059174	POST>PRE	ENSG00000213362	FTH1P12	0.00062235	1.646771486	PRE>POST
ENSG00000100316	RPL3	0.000481108	-1.050939894	POST>PRE	ENSG00000145908	ZNF300	3.97E-05	1.644095814	PRE>POST
ENSG00000163382	APOA1BP	0.000312119	-1.050919229	POST>PRE	ENSG00000203914	HSP90B3P	0.000590737	1.643516953	PRE>POST
ENSG00000114391	RPL24	0.000514079	-1.050824402	POST>PRE	ENSG00000256356	HSPA8P5	9.37E-06	1.638645163	PRE>POST
ENSG00000165283	STOML2	0.000407729	-1.050786769	POST>PRE	ENSG00000182263	FIGN	0.000929571	1.635245729	PRE>POST
ENSG00000169223	LMAN2	0.000160354	-1.050360229	POST>PRE	ENSG00000268670	CTD-2622I13,3	0.00062814	1.629735053	PRE>POST
ENSG00000108298	RPL19	9.79E-06	-1.050237534	POST>PRE	ENSG00000243859	RPL5P17	4.74E-07	1.60889734	PRE>POST
ENSG00000136715	SAP130	0.000570421	-1.050023105	POST>PRE	ENSG00000047617	ANO2	0.000225761	1.60617317	PRE>POST
ENSG00000069275	NUCKS1	7.30E-05	-1.049979011	POST>PRE	ENSG00000105808	RASA4	0.000929366	1.605981181	PRE>POST
ENSG00000137055	PLAA	0.000835138	-1.049744589	POST>PRE	ENSG00000269688	AC008982,2	0.000731481	1.604966949	PRE>POST
ENSG00000134825	TMEM258	4.19E-05	-1.04970687	POST>PRE	ENSG00000235605	RP5-827C21,1	3.16E-06	1.603687272	PRE>POST

ENSG00000135829	DHX9	0.000419597	-1.049700228	POST>PRE	ENSG00000128739	SNRPN	0.000168216	1.594568011	PRE>POST
ENSG00000168724	DNAJC21	0.000241072	-1.049698423	POST>PRE	ENSG00000224376	AC017104,6	0.000355264	1.59086896	PRE>POST
ENSG00000145592	RPL37	0.000207062	-1.049572176	POST>PRE	ENSG00000203709	C1ORF132	6.22E-07	1.564335212	PRE>POST
ENSG00000105323	HNRNPUL1	8.54E-05	-1.0493967	POST>PRE	ENSG00000188873	RPL10AP2	6.13E-07	1.560402916	PRE>POST
ENSG00000131473	ACLY	0.000176353	-1.049015517	POST>PRE	ENSG00000213763	ACTBP2	6.19E-08	1.557775507	PRE>POST
ENSG00000133265	HSPBP1	0.000831764	-1.048788489	POST>PRE	ENSG00000131080	EDA2R	8.29E-05	1.54909935	PRE>POST
ENSG00000113384	GOLPH3	0.000299669	-1.048605963	POST>PRE	ENSG00000271553	RP11-274B21,10	0.0004559	1.546772618	PRE>POST
ENSG00000131469	RPL27	9.36E-06	-1.048547754	POST>PRE	ENSG00000262194	CTD-3195I5,5	0.000977745	1.541440221	PRE>POST
ENSG00000153187	HNRNPU	0.000124991	-1.048467339	POST>PRE	ENSG00000177599	ZNF491	2.13E-05	1.535608123	PRE>POST
ENSG00000141552	ANAPC11	0.00061712	-1.048226463	POST>PRE	ENSG00000261655	CTD-3064M3,3	2.09E-05	1.533285599	PRE>POST
ENSG00000149925	ALDOA	0.000774661	-1.04813351	POST>PRE	ENSG00000265206	MIR142	0.000363452	1.522944204	PRE>POST
ENSG00000142864	SERBP1	0.000492543	-1.048124599	POST>PRE	ENSG00000268032	CTB-52I2,4	2.00E-05	1.521964462	PRE>POST
ENSG00000159111	MRPL10	8.49E-05	-1.048068189	POST>PRE	ENSG00000152154	TMEM178A	0.000409428	1.515183533	PRE>POST
ENSG00000197756	RPL37A	0.000384083	-1.047830278	POST>PRE	ENSG00000268006	PTOV1-AS1	0.000134205	1.511770511	PRE>POST
ENSG00000089009	RPL6	0.000409287	-1.047619316	POST>PRE	ENSG00000149451	ADAM33	0.000553032	1.511353512	PRE>POST
ENSG00000177600	RPLP2	9.35E-06	-1.047281551	POST>PRE	ENSG00000240509	RPL34P18	6.62E-07	1.508470336	PRE>POST
ENSG00000188612	SUMO2	1.55E-07	-1.047052651	POST>PRE	ENSG00000218208	RP11-367G18,2	0.000750422	1.499196316	PRE>POST
ENSG00000165264	NDUFB6	0.000691906	-1.04682263	POST>PRE	ENSG00000235587	GAPDHP65	4.45E-07	1.490404798	PRE>POST
ENSG00000168883	USP39	0.000966631	-1.046720808	POST>PRE	ENSG00000167371	PRRT2	7.86E-05	1.487777473	PRE>POST
ENSG00000079246	XRCC5	5.92E-06	-1.046620081	POST>PRE	ENSG00000242028	HYPK	5.34E-06	1.487612657	PRE>POST
ENSG00000175467	SART1	0.000259486	-1.046485194	POST>PRE	ENSG00000237781	RP11-54A4,2	0.000926355	1.4864352	PRE>POST
ENSG00000172809	RPL38	0.000188901	-1.046481123	POST>PRE	ENSG00000157306	RP11-66N24,4	0.000120341	1.485504179	PRE>POST
ENSG00000108883	EFTUD2	0.000398193	-1.046375161	POST>PRE	ENSG00000131055	COX4I2	0.000267912	1.48270852	PRE>POST
ENSG00000145907	G3BP1	0.00077919	-1.046325346	POST>PRE	ENSG00000130307	USHBP1	0.000176035	1.479627386	PRE>POST
ENSG00000125944	HNRNPR	0.000417306	-1.046218352	POST>PRE	ENSG00000250506	CDK3	0.000235031	1.466359901	PRE>POST
ENSG00000143621	ILF2	0.000311045	-1.046111099	POST>PRE	ENSG00000255837	TAS2R20	5.59E-05	1.465702138	PRE>POST
ENSG00000109475	RPL34	0.000509775	-1.045708639	POST>PRE	ENSG00000264672	RP11-112H10,4	0.000529598	1.465231938	PRE>POST
ENSG00000180329	CCDC43	0.000743453	-1.045452164	POST>PRE	ENSG00000143127	ITGA10	4.72E-05	1.462528189	PRE>POST
ENSG00000137656	BUD13	0.000512301	-1.045404798	POST>PRE	ENSG00000224550	RP11-270C12,3	8.52E-05	1.461340732	PRE>POST
ENSG00000122705	CLTA	0.000163777	-1.044978627	POST>PRE	ENSG00000203392	AC105020,1	0.000136961	1.459277853	PRE>POST

ENSG00000213719	CLIC1	0.000304615	-1.044818118	POST>PRE	ENSG00000250539	KRT8P33	9.15E-05	1.45764195	PRE>POST
ENSG00000116199	FAM20B	0.000160567	-1.044691925	POST>PRE	ENSG00000162367	TAL1	0.00018197	1.456269227	PRE>POST
ENSG00000198356	ASNA1	0.000843763	-1.044671902	POST>PRE	ENSG00000144596	GRIP2	0.000527763	1.455781116	PRE>POST
ENSG00000108278	ZNHIT3	3.08E-05	-1.044629768	POST>PRE	ENSG00000229719	MIR194-2	0.000292723	1.443008	PRE>POST
ENSG00000131507	NDFIP1	0.000457486	-1.044201296	POST>PRE	ENSG00000257151	PWAR6	0.000758808	1.44064774	PRE>POST
ENSG00000163510	CWC22	0.000371939	-1.044090095	POST>PRE	ENSG00000157103	SLC6A1	4.72E-05	1.43902409	PRE>POST
ENSG00000257103	LSM14A	1.71E-05	-1.043887324	POST>PRE	ENSG00000254285	KRT8P3	0.00013042	1.43546897	PRE>POST
ENSG00000142676	RPL11	9.60E-05	-1.043738692	POST>PRE	ENSG00000251992	SCARNA17	0.000984057	1.433219253	PRE>POST
ENSG00000134308	YWHAQ	0.000877517	-1.043721142	POST>PRE	ENSG00000213613	RP11-380G5,3	0.000519377	1.432181379	PRE>POST
ENSG00000077312	SNRPA	0.000911537	-1.043425956	POST>PRE	ENSG00000117425	PTCH2	0.000641388	1.431632024	PRE>POST
ENSG00000079999	KEAP1	0.000159345	-1.043167419	POST>PRE	ENSG00000226221	AC022431,1	8.31E-05	1.420893451	PRE>POST
ENSG00000113141	IK	0.000892622	-1.04311738	POST>PRE	ENSG00000269936	MIR145	8.94E-05	1.41956185	PRE>POST
ENSG00000132604	TERF2	0.000162639	-1.042993318	POST>PRE	ENSG00000233830	EIF4HP1	6.18E-06	1.417920799	PRE>POST
ENSG00000143740	SNAP47	0.000755643	-1.042901441	POST>PRE	ENSG00000180211	RP1-278E11,3	1.01E-05	1.416146731	PRE>POST
ENSG00000233927	RPS28	0.000966395	-1.042495092	POST>PRE	ENSG00000239653	PSMD6-AS2	1.95E-05	1.41257198	PRE>POST
ENSG00000167986	DDB1	0.000106971	-1.042464715	POST>PRE	ENSG00000259529	RP11-468E2,4	0.000206501	1.410348964	PRE>POST
ENSG00000169714	CNBP	0.000418582	-1.042383854	POST>PRE	ENSG00000197182	FLJ27365	4.65E-07	1.409478793	PRE>POST
ENSG00000099817	POLR2E	7.79E-06	-1.041811737	POST>PRE	ENSG00000204253	HNRNPCP2	4.23E-05	1.40325789	PRE>POST
ENSG00000177954	RPS27	0.000551165	-1.041698687	POST>PRE	ENSG00000240288	GHRLOS	2.81E-05	1.400379597	PRE>POST
ENSG00000177830	CHID1	0.000520187	-1.041583468	POST>PRE	ENSG00000214827	MTCP1	2.99E-05	1.399299792	PRE>POST
ENSG00000118217	ATF6	0.000343318	-1.041189382	POST>PRE	ENSG00000254373	RP11-34P1,2	0.000283776	1.3986528	PRE>POST
ENSG00000048649	RSF1	0.000257573	-1.041175144	POST>PRE	ENSG00000253676	TAGLN2P1	0.000632678	1.391753798	PRE>POST
ENSG00000169230	PRELID1	6.78E-05	-1.041087116	POST>PRE	ENSG00000214548	MEG3	1.20E-06	1.388846082	PRE>POST
ENSG00000111640	GAPDH	0.000106185	-1.040487969	POST>PRE	ENSG00000231043	AC007238,1	0.000961621	1.388036929	PRE>POST
ENSG00000196531	NACA	0.000832456	-1.040402679	POST>PRE	ENSG00000250510	GPR162	0.000453213	1.386777076	PRE>POST
ENSG00000103429	BFAR	0.000759876	-1.040364209	POST>PRE	ENSG00000152527	PLEKHH2	6.29E-06	1.383847643	PRE>POST
ENSG00000089597	GANAB	3.87E-05	-1.04028365	POST>PRE	ENSG00000147437	GNRH1	1.51E-05	1.376936416	PRE>POST
ENSG00000161021	MAML1	0.000727118	-1.03924838	POST>PRE	ENSG00000236801	RPL24P8	4.20E-05	1.375095525	PRE>POST
ENSG00000211460	TSN	0.000556543	-1.039220016	POST>PRE	ENSG00000229119	CTB-63M22,1	1.27E-06	1.372647202	PRE>POST
ENSG00000221983	UBA52	0.000336573	-1.038934019	POST>PRE	ENSG00000067445	TRO	9.27E-05	1.369382736	PRE>POST

ENSG00000121774	KHDRBS1	7.88E-05	-1.038547129	POST>PRE
ENSG00000113387	SUB1	0.00039714	-1.038494126	POST>PRE
ENSG00000067596	DHX8	0.00015344	-1.038473222	POST>PRE
ENSG00000133112	TPT1	0.000786619	-1.038346241	POST>PRE
ENSG00000069345	DNAJA2	0.000967591	-1.038254119	POST>PRE
ENSG00000182934	SRPR	0.000616426	-1.03816122	POST>PRE
ENSG00000175582	RAB6A	5.38E-05	-1.037768715	POST>PRE
ENSG00000009307	CSDE1	0.000463896	-1.037121916	POST>PRE
ENSG00000143612	C1ORF43	1.31E-05	-1.037046388	POST>PRE
ENSG00000165006	UBAP1	0.000160374	-1.036797887	POST>PRE
ENSG00000087365	SF3B2	1.54E-05	-1.036692247	POST>PRE
ENSG00000122692	SMU1	6.90E-05	-1.036538815	POST>PRE
ENSG00000120265	PCMT1	0.000551691	-1.03636189	POST>PRE
ENSG00000011304	PTBP1	0.000383238	-1.036069083	POST>PRE
ENSG00000108344	PSMD3	0.000476727	-1.03449371	POST>PRE
ENSG00000108294	PSMB3	0.000929564	-1.033852581	POST>PRE
ENSG00000173039	RELA	6.21E-05	-1.033451496	POST>PRE
ENSG00000173812	EIF1	0.000464182	-1.033158183	POST>PRE
ENSG00000104824	HNRNPL	0.000303812	-1.030958648	POST>PRE
ENSG00000099341	PSMD8	0.000590278	-1.03061226	POST>PRE
ENSG00000099783	HNRNPM	0.000246511	-1.030366875	POST>PRE

ENSG00000260778	MIR940	0.000395382	1.369147275	PRE>POST
ENSG00000231234	SKP1P1	2.95E-05	1.369065364	PRE>POST
ENSG00000272040	CTC-366B18,4	0.000497599	1.368319941	PRE>POST
ENSG00000225356	RP11-433O3,1	0.000927544	1.367095938	PRE>POST
ENSG00000272661	RP4-548D19,3	0.000328425	1.364029646	PRE>POST
ENSG00000243964	RP11-16F15,1	0.00036421	1.362353733	PRE>POST
ENSG00000092529	CAPN3	6.80E-05	1.362209	PRE>POST
ENSG00000239763	AC009120,3	0.000861478	1.359967272	PRE>POST
ENSG00000166343	MSS51	5.74E-09	1.356749197	PRE>POST
ENSG00000254505	CHMP4A	0.000651732	1.351126471	PRE>POST
ENSG00000180190	TDRP	0.000140332	1.350016713	PRE>POST
ENSG00000142303	ADAMTS10	0.000312761	1.349705396	PRE>POST
ENSG00000135740	SLC9A5	0.000695071	1.348929362	PRE>POST
ENSG00000243547	HNRNPKP4	2.63E-06	1.344220214	PRE>POST
ENSG00000213846	AC098614,2	0.000259947	1.342134064	PRE>POST
ENSG00000129749	CHRNA10	0.000372237	1.34125108	PRE>POST
ENSG00000224415	RPL19P12	9.79E-06	1.335416523	PRE>POST
ENSG00000229645	LINC00341	0.000203392	1.334859916	PRE>POST
ENSG00000264456	RP11-848P1,2	0.000616271	1.334651399	PRE>POST
ENSG00000224975	INE1	0.000127225	1.333941223	PRE>POST
ENSG00000267940	RP11-290F24,6	0.000425369	1.331502426	PRE>POST
ENSG00000248019	FAM13A-AS1	0.000106077	1.326313134	PRE>POST
ENSG00000222009	BTBD19	0.000833062	1.324431918	PRE>POST
ENSG00000129467	ADCY4	3.29E-05	1.323952026	PRE>POST
ENSG00000186594	MIR22HG	7.28E-05	1.317470707	PRE>POST
ENSG00000220785	MTMR9LP	6.15E-06	1.314898626	PRE>POST
ENSG00000214279	RP11-108K14,4	0.00051919	1.314706364	PRE>POST
ENSG00000261490	RP11-448G15,3	2.31E-06	1.314310202	PRE>POST
ENSG00000241014	RP11-244H3,1	4.14E-07	1.310501793	PRE>POST
ENSG00000172748	ZNF596	7.73E-06	1.306848961	PRE>POST

ENSG00000235316	DUSP8P5	0.000349697	1.303702952	PRE>POST
ENSG00000232176	RP11-146N23,1	7.78E-05	1.300814618	PRE>POST
ENSG00000246922	UBAP1L	4.14E-05	1.297180648	PRE>POST
ENSG00000225828	FAM229A	4.18E-05	1.297036429	PRE>POST
ENSG00000181524	RPL24P4	3.16E-05	1.294033129	PRE>POST
ENSG00000219023	RP3-340B19,2	0.000802452	1.288948728	PRE>POST
ENSG00000236901	MIR600HG	0.000153795	1.288938673	PRE>POST
ENSG00000260793	RP5-882C2,2	0.000524547	1.287968005	PRE>POST
ENSG00000249264	EEF1A1P9	2.74E-05	1.287712582	PRE>POST
ENSG00000197776	KLHDC1	0.00013417	1.287667646	PRE>POST
ENSG00000166532	RIMKLB	0.000181287	1.287358755	PRE>POST
ENSG00000262903	RP11-235E17,6	4.84E-05	1.283837708	PRE>POST
ENSG00000261324	RP11-174G6,5	0.000442926	1.283532977	PRE>POST
ENSG00000267283	AC005306,3	0.00014026	1.282353228	PRE>POST
ENSG00000258727	RP11-66N24,3	5.52E-06	1.281192746	PRE>POST
ENSG00000213598	RP11-112J1,1	0.000313591	1.280647628	PRE>POST
ENSG00000212127	TAS2R14	0.000115224	1.274026496	PRE>POST
ENSG00000255031	RP11-802E16,3	0.000418569	1.273257055	PRE>POST
ENSG00000273449	RP11-218F10,3	2.66E-06	1.273183255	PRE>POST
ENSG00000161381	PLXDC1	9.21E-05	1.270883975	PRE>POST
ENSG00000224660	SH3BP5-AS1	0.000405741	1.269565235	PRE>POST
ENSG00000138316	ADAMTS14	0.000139657	1.266373598	PRE>POST
ENSG00000221995	TIAF1	2.11E-05	1.265751578	PRE>POST
ENSG00000135407	AVIL	3.21E-06	1.262596413	PRE>POST
ENSG00000240291	RP11-499P20,2	0.000418001	1.259444286	PRE>POST
ENSG00000259706	HSP90B2P	0.000371313	1.259063949	PRE>POST
ENSG00000213139	CRYGS	5.59E-05	1.257229996	PRE>POST
ENSG00000215769	MIR6080	0.000183154	1.255609678	PRE>POST
ENSG00000213398	LCAT	0.000464183	1.255168298	PRE>POST
ENSG00000121068	TBX2	0.000129923	1.249277447	PRE>POST

ENSG00000244701	RP5-894A10,2	9.52E-05	1.248247621	PRE>POST
ENSG00000234782	TPT1P9	0.000100383	1.24339471	PRE>POST
ENSG00000224861	YBX1P1	0.000866169	1.243101135	PRE>POST
ENSG00000261094	RP11-355O1,11	4.85E-05	1.242409296	PRE>POST
ENSG00000272356	RP5-1112D6,8	0.000230553	1.241074447	PRE>POST
ENSG00000181016	LSMEM1	0.000176054	1.238936817	PRE>POST
ENSG00000132522	GPS2	7.84E-05	1.238361166	PRE>POST
ENSG00000223768	LINC00205	0.000694187	1.238320456	PRE>POST
ENSG00000256745	RP11-680H20,1	0.00096122	1.23759135	PRE>POST
ENSG00000204305	AGER	0.000167875	1.236711684	PRE>POST
ENSG00000224631	RP11-51O6,1	3.17E-09	1.236217913	PRE>POST
ENSG00000114654	EFCC1	0.000775882	1.234690815	PRE>POST
ENSG00000215067	AC027763,2	0.000581189	1.234356794	PRE>POST
ENSG00000232931	LINC00342	0.00019834	1.231424734	PRE>POST
ENSG00000259972	AC009120,6	0.00010452	1.230324304	PRE>POST
ENSG00000250959	GLUD1P3	0.000212122	1.22872369	PRE>POST
ENSG00000272807	RP11-260M2,1	0.000173871	1.228490929	PRE>POST
ENSG00000218175	AC016739,2	1.96E-05	1.227678958	PRE>POST
ENSG00000267796	LIN37	0.0004332	1.220689445	PRE>POST
ENSG00000198695	MT-ND6	0.000356604	1.220098612	PRE>POST
ENSG00000167555	ZNF528	0.000364289	1.219740591	PRE>POST
ENSG00000232637	WI2-3658N16,1	0.000133859	1.218777591	PRE>POST
ENSG00000264469	RP11-173M1,8	0.000105079	1.215648487	PRE>POST
ENSG00000100968	NFATC4	0.000510788	1.211477348	PRE>POST
ENSG00000271430	RP3-368A4,5	5.75E-05	1.205118713	PRE>POST
ENSG00000169129	AFAP1L2	0.00062053	1.204417221	PRE>POST
ENSG00000228232	GAPDHP1	0.000130448	1.204405054	PRE>POST
ENSG00000258441	LINC00641	5.56E-05	1.202307797	PRE>POST
ENSG00000174483	BBS1	0.000569441	1.202168399	PRE>POST
ENSG00000181619	GPR135	0.00063264	1.201414652	PRE>POST

ENSG00000239779	WBP1	0.000534236	1.200935594	PRE>POST
ENSG00000273131	RP6-42F4,1	0.000440789	1.2003095	PRE>POST
ENSG00000272141	RP11-465B22,8	0.000580396	1.199168252	PRE>POST
ENSG00000261326	RP5-1057J7,6	0.000441125	1.19895438	PRE>POST
ENSG00000212694	AC084018,1	4.51E-05	1.196874963	PRE>POST
ENSG00000266173	STRADA	0.000104038	1.196557	PRE>POST
ENSG00000249592	RP11-440L14,1	1.86E-05	1.196429713	PRE>POST
ENSG00000075826	SEC31B	0.000557702	1.195901041	PRE>POST
ENSG00000217930	PAM16	8.36E-05	1.195143008	PRE>POST
ENSG00000175265	GOLGA8A	0.000292639	1.193055117	PRE>POST
ENSG00000111215	PRR4	0.000679632	1.192794817	PRE>POST
ENSG00000204681	GABBR1	0.000417728	1.190562597	PRE>POST
ENSG00000100288	CHKB	0.000552067	1.18345336	PRE>POST
ENSG00000100767	PAPLN	0.00092712	1.179279684	PRE>POST
ENSG00000196696	PDXDC2P	0.000484141	1.177800693	PRE>POST
ENSG00000186275	NBPF12	0.000117302	1.17520423	PRE>POST
ENSG00000165359	DDX26B	0.000759922	1.17261901	PRE>POST
ENSG00000163463	KRTCAP2	4.21E-05	1.17123352	PRE>POST
ENSG00000271533	RP3-368A4,6	0.000629351	1.16945625	PRE>POST
ENSG00000239665	RP11-295P9,3	1.87E-06	1.166590908	PRE>POST
ENSG00000198786	MT-ND5	2.21E-05	1.165917029	PRE>POST
ENSG00000179532	DNHD1	0.000532795	1.164817148	PRE>POST
ENSG00000186814	ZSCAN30	0.000254251	1.163866511	PRE>POST
ENSG00000122390	NAA60	0.000625789	1.161742936	PRE>POST
ENSG00000148824	MTG1	0.000402618	1.158803308	PRE>POST
ENSG00000205885	C1RL-AS1	0.000793114	1.154691306	PRE>POST
ENSG00000206149	HERC2P9	1.29E-06	1.154535211	PRE>POST
ENSG00000019144	PHLDB1	0.000454405	1.150597938	PRE>POST
ENSG00000198888	MT-ND1	0.000729272	1.146270348	PRE>POST
ENSG00000179979	CRIPAK	3.57E-05	1.145992683	PRE>POST

ENSG00000269352	AC018766,5	0.000975666	1.144982445	PRE>POST
ENSG00000139631	CSAD	6.91E-05	1.144257184	PRE>POST
ENSG00000196205	EEF1A1P5	0.000127181	1.141721922	PRE>POST
ENSG00000251562	MALAT1	1.05E-05	1.139906534	PRE>POST
ENSG00000156345	CDK20	0.000596647	1.139521637	PRE>POST
ENSG00000163945	UVSSA	0.000454914	1.138597196	PRE>POST
ENSG00000259820	AC083843,1	0.000205726	1.137464792	PRE>POST
ENSG00000126790	L3HYPDH	0.000274918	1.136338931	PRE>POST
ENSG00000204149	AGAP6	6.75E-05	1.13484342	PRE>POST
ENSG00000214021	TTLL3	0.000760724	1.13353683	PRE>POST
ENSG00000259865	RP11-488L18,10	0.000344153	1.133018126	PRE>POST
ENSG00000174093	RP11-1407O15,2	5.35E-05	1.13194045	PRE>POST
ENSG00000182685	BRICD5	0.000405849	1.129923079	PRE>POST
ENSG00000186166	CCDC84	3.97E-05	1.129343611	PRE>POST
ENSG00000175787	ZNF169	3.11E-05	1.128190686	PRE>POST
ENSG00000230715	RP11-274B21,4	0.00068351	1.127323489	PRE>POST
ENSG00000169246	NPIPB3	0.000459379	1.125991381	PRE>POST
ENSG00000204427	ABHD16A	0.000518442	1.1243754	PRE>POST
ENSG00000260565	ERVK13-1	0.000833837	1.12258058	PRE>POST
ENSG00000154803	FLCN	0.000277592	1.120658501	PRE>POST
ENSG00000166436	TRIM66	1.01E-05	1.120321771	PRE>POST
ENSG00000157036	EXOG	4.70E-05	1.118637119	PRE>POST
ENSG00000269858	EGLN2	7.35E-05	1.118059015	PRE>POST
ENSG00000173209	AHSA2	9.11E-05	1.11805223	PRE>POST
ENSG00000196123	KIAA0895L	0.000938323	1.115623502	PRE>POST
ENSG00000179818	PCBP1-AS1	0.000313302	1.113537339	PRE>POST
ENSG00000168010	ATG16L2	0.000737575	1.110442387	PRE>POST
ENSG00000100483	VCPKMT	0.000510097	1.109658056	PRE>POST
ENSG00000117569	PTBP2	0.000361803	1.108692847	PRE>POST
ENSG00000142396	ERVK3-1	0.00058101	1.107257219	PRE>POST

ENSG00000113971	NPHP3	0.000611101	1.107116653	PRE>POST
ENSG00000114770	ABCC5	4.94E-05	1.106836265	PRE>POST
ENSG00000269958	RP11-73M18,8	0.000455014	1.106730946	PRE>POST
ENSG00000110888	CAPRIN2	0.000537445	1.106707881	PRE>POST
ENSG00000047056	WDR37	5.49E-05	1.104355048	PRE>POST
ENSG00000175309	PHYKPL	0.000698202	1.10388699	PRE>POST
ENSG00000186866	POFUT2	0.000533472	1.103784721	PRE>POST
ENSG00000121310	ECHDC2	0.000217768	1.102915737	PRE>POST
ENSG00000215908	CROCCP2	8.91E-05	1.102678783	PRE>POST
ENSG00000163867	ZMYM6	4.50E-05	1.101895427	PRE>POST
ENSG00000228253	MT-ATP8	0.000246532	1.101830655	PRE>POST
ENSG00000262758	CTD-3195I5,1	5.76E-05	1.10108154	PRE>POST
ENSG00000115459	ELMOD3	2.80E-05	1.100850079	PRE>POST
ENSG00000198804	MT-CO1	0.000759696	1.100135634	PRE>POST
ENSG00000120458	MSANTD2	0.000930709	1.099491661	PRE>POST
ENSG00000248527	MTATP6P1	0.000167389	1.098144568	PRE>POST
ENSG00000257621	RP11-349A22,5	1.85E-05	1.097875119	PRE>POST
ENSG00000126214	KLC1	8.54E-06	1.097621183	PRE>POST
ENSG00000184465	WDR27	0.000493403	1.097471081	PRE>POST
ENSG00000184787	UBE2G2	0.000604225	1.094742901	PRE>POST
ENSG00000131584	ACAP3	0.000355745	1.094543452	PRE>POST
ENSG00000267680	ZNF224	0.00058931	1.094189421	PRE>POST
ENSG00000268205	CTC-444N24,11	0.000288175	1.09211838	PRE>POST
ENSG00000134884	ARGLU1	0.000537499	1.09170335	PRE>POST
ENSG00000109046	WSB1	0.000616331	1.088432689	PRE>POST
ENSG00000122912	SLC25A16	0.000181783	1.08813812	PRE>POST
ENSG00000227671	MIR3916	0.000563667	1.088061323	PRE>POST
ENSG00000135164	DMTF1	1.35E-05	1.086949832	PRE>POST
ENSG00000258634	RP4-773N10,4	0.000492936	1.086646124	PRE>POST
ENSG00000139636	LMBR1L	0.000108827	1.085923001	PRE>POST

ENSG00000198105	ZNF248	0.000513058	1.085870301	PRE>POST
ENSG00000119688	ABCD4	0.00016761	1.08544085	PRE>POST
ENSG00000185246	PRPF39	2.34E-05	1.08512577	PRE>POST
ENSG00000212907	MT-ND4L	0.000928199	1.084756647	PRE>POST
ENSG00000160613	PCSK7	0.000628453	1.084559812	PRE>POST
ENSG00000168096	ANKS3	0.000234593	1.084228746	PRE>POST
ENSG00000114857	NKTR	5.86E-05	1.082136532	PRE>POST
ENSG00000163386	NBPF10	0.000675137	1.082031773	PRE>POST
ENSG00000113240	CLK4	0.000424779	1.081991762	PRE>POST
ENSG00000198899	MT-ATP6	0.000189172	1.080776516	PRE>POST
ENSG00000102901	CENPT	0.000304796	1.080334363	PRE>POST
ENSG00000068885	IFT80	0.000454397	1.080149632	PRE>POST
ENSG00000013441	CLK1	1.30E-05	1.0792221	PRE>POST
ENSG00000088448	ANKRD10	0.000367686	1.079219884	PRE>POST
ENSG00000061987	MON2	1.06E-05	1.078175993	PRE>POST
ENSG00000108669	CYTH1	0.00056535	1.077707281	PRE>POST
ENSG00000198625	MDM4	4.33E-05	1.076983328	PRE>POST
ENSG00000074356	C17ORF85	0.000159521	1.07682993	PRE>POST
ENSG00000131791	PRKAB2	5.93E-06	1.076651925	PRE>POST
ENSG00000204130	RUFY2	0.000173884	1.076250253	PRE>POST
ENSG00000003756	RBM5	5.21E-06	1.075728217	PRE>POST
ENSG00000198886	MT-ND4	0.000456827	1.073720976	PRE>POST
ENSG00000223959	AFG3L1P	0.00018049	1.07322449	PRE>POST
ENSG00000158805	ZNF276	0.000213904	1.072855669	PRE>POST
ENSG00000165792	METTL17	0.000928156	1.072070034	PRE>POST
ENSG00000198198	SZT2	0.000134161	1.068756447	PRE>POST
ENSG00000205356	TECPR1	0.000729316	1.068409716	PRE>POST
ENSG00000053900	ANAPC4	5.81E-07	1.068131951	PRE>POST
ENSG00000172613	RAD9A	0.00055597	1.067975427	PRE>POST
ENSG00000116001	TIA1	4.14E-05	1.067772003	PRE>POST

ENSG00000132424	PNISR	0.000196291	1.067467309	PRE>POST
ENSG00000167528	ZNF641	0.000344723	1.067190155	PRE>POST
ENSG00000010322	NISCH	4.84E-05	1.067041706	PRE>POST
ENSG00000100650	SRSF5	1.96E-05	1.065692033	PRE>POST
ENSG00000153914	SREK1	0.000428379	1.064116563	PRE>POST
ENSG00000130023	ERMARD	0.000435749	1.063613601	PRE>POST
ENSG00000163660	CCNL1	5.13E-05	1.062949266	PRE>POST
ENSG00000204084	INPP5B	0.000608781	1.062287641	PRE>POST
ENSG00000082258	CCNT2	0.000611761	1.06096917	PRE>POST
ENSG00000165699	TSC1	0.000969065	1.06001682	PRE>POST
ENSG00000116497	S100PBP	0.000312112	1.059493304	PRE>POST
ENSG00000164073	MFSD8	0.000370087	1.059436318	PRE>POST
ENSG00000215041	NEURL4	0.000619514	1.059359564	PRE>POST
ENSG00000169155	ZBTB43	0.000968555	1.058506287	PRE>POST
ENSG00000176444	CLK2	0.000507938	1.058423035	PRE>POST
ENSG00000128699	ORMDL1	2.44E-06	1.057845529	PRE>POST
ENSG00000263244	RP11-473I1,10	5.73E-08	1.057324563	PRE>POST
ENSG00000155903	RASA2	0.000728538	1.055768855	PRE>POST
ENSG00000090905	TNRC6A	0.000633046	1.052264151	PRE>POST
ENSG00000119707	RBM25	0.000177479	1.051190135	PRE>POST
ENSG00000143442	POGZ	2.81E-05	1.050752331	PRE>POST
ENSG00000136147	PHF11	0.000352653	1.050091734	PRE>POST
ENSG00000135945	REV1	0.000316579	1.049836031	PRE>POST
ENSG00000197912	SPG7	0.000473963	1.048232667	PRE>POST
ENSG00000158411	MITD1	0.000892515	1.047734832	PRE>POST
ENSG00000126804	ZBTB1	0.000259088	1.046890073	PRE>POST
ENSG00000122085	MTERF4	0.000408016	1.046809125	PRE>POST
ENSG00000186001	LRCH3	3.60E-06	1.045854049	PRE>POST
ENSG00000108654	DDX5	6.02E-05	1.04489053	PRE>POST
ENSG00000169045	HNRNPH1	0.000509716	1.043936005	PRE>POST

ENSG00000100201	DDX17	0.000221642	1.043930579	PRE>POST
ENSG00000119760	SUPT7L	0.000975463	1.04283571	PRE>POST
ENSG00000162231	NXF1	0.000402067	1.040542132	PRE>POST
ENSG00000149930	TAOK2	0.000217158	1.037641042	PRE>POST
ENSG00000198408	MGEA5	0.000835622	1.03511337	PRE>POST

aThis analysis was performed using the R2 omics data analysis platform using a differentially gene expression analysis (ANOVA; FDR <0.001). Gene lists are sorted by the magnitude of fold-change.

Imatinib

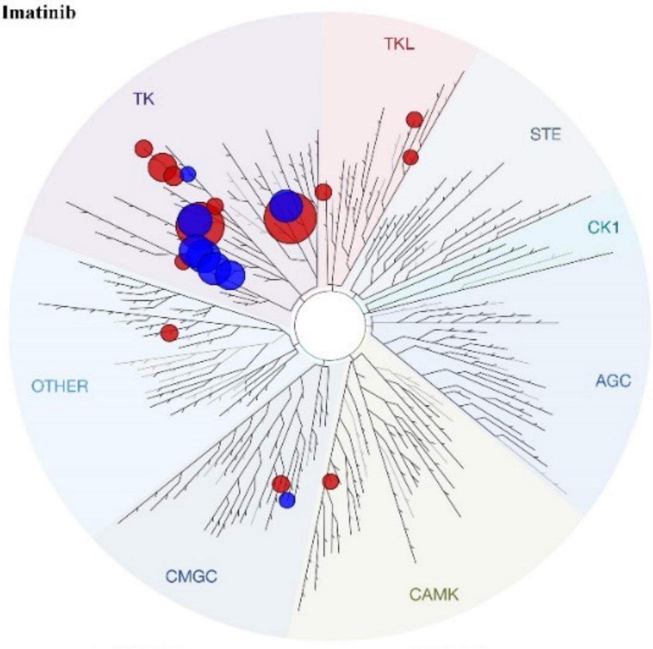

Supplemental Figure S1. Imatinib is a selective inhibitor of tyrosine kinases overexpressed in CMS4 CRC. The activity spectrum of imatinib was mapped on the human kinome. The size of each dot reflects the IC50 of each drug for specific kinases. Larger spots indicate lower IC50s and more efficient inhibition. Imatinib targets that are significantly overexpressed in CMS4 CRC (See Table S4) are highlighted in blue. All other imatinib targets are in red. The image was generated using the TREEspot™ software tool and reprinted with permission from KINOMEscan®, a division of DiscoveRx Corporation, © DISCOVERX CORPORATION 2010.

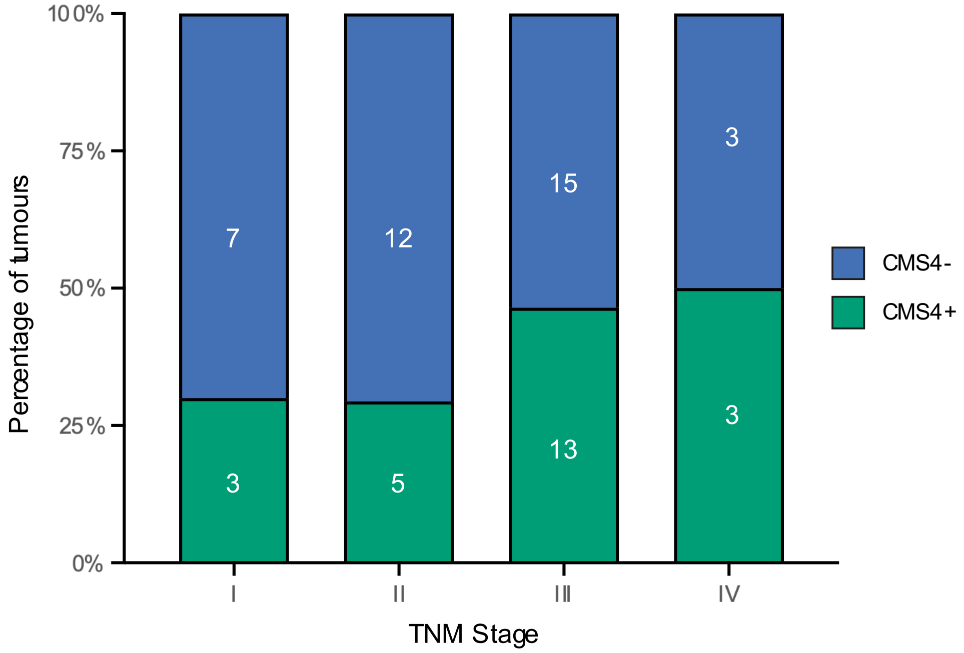

Supplemental Figure S2. Bar graph showing the incidence of CMS4 across CRC AJCC TNM stages in the ImPACCT cohort.

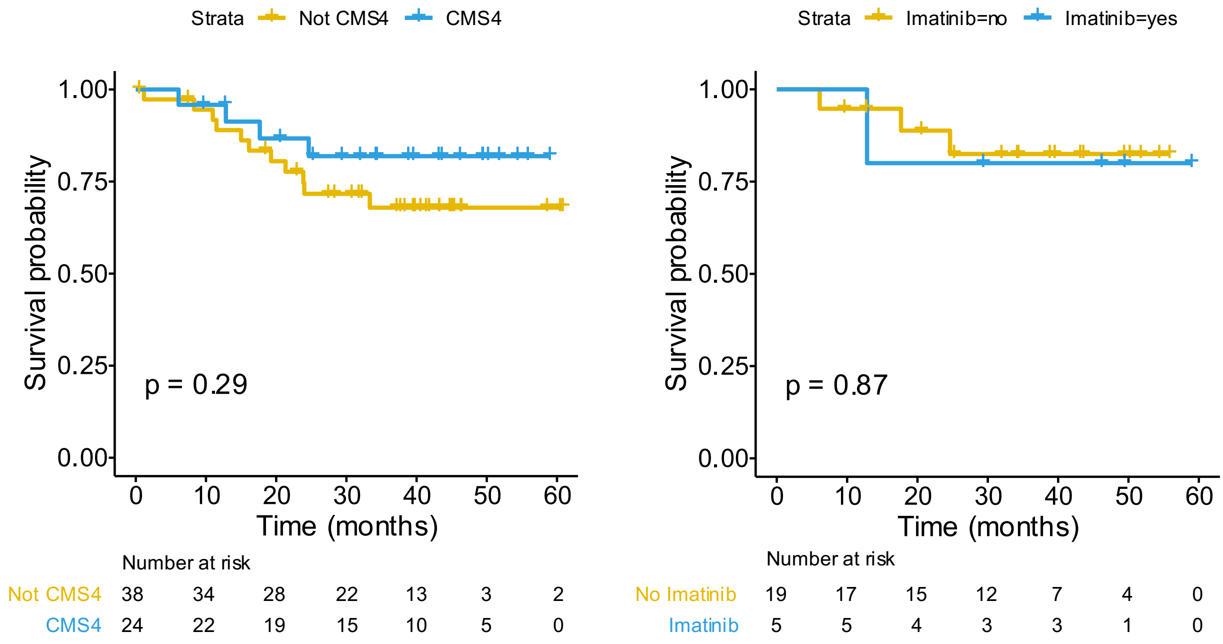

Supplemental Figure S3. Kaplan Meier survival estimate plots demonstrating overall survival of CRC patients in the ImPACCT cohort. The left panel shows overall survival of patients with CMS4 and non-CMS4 tumors. The right panel shows overall survival of patients with CMS4 tumors who received imatinib treatment *versus* those who did not. Survival distributions were compared using the log-rank test.

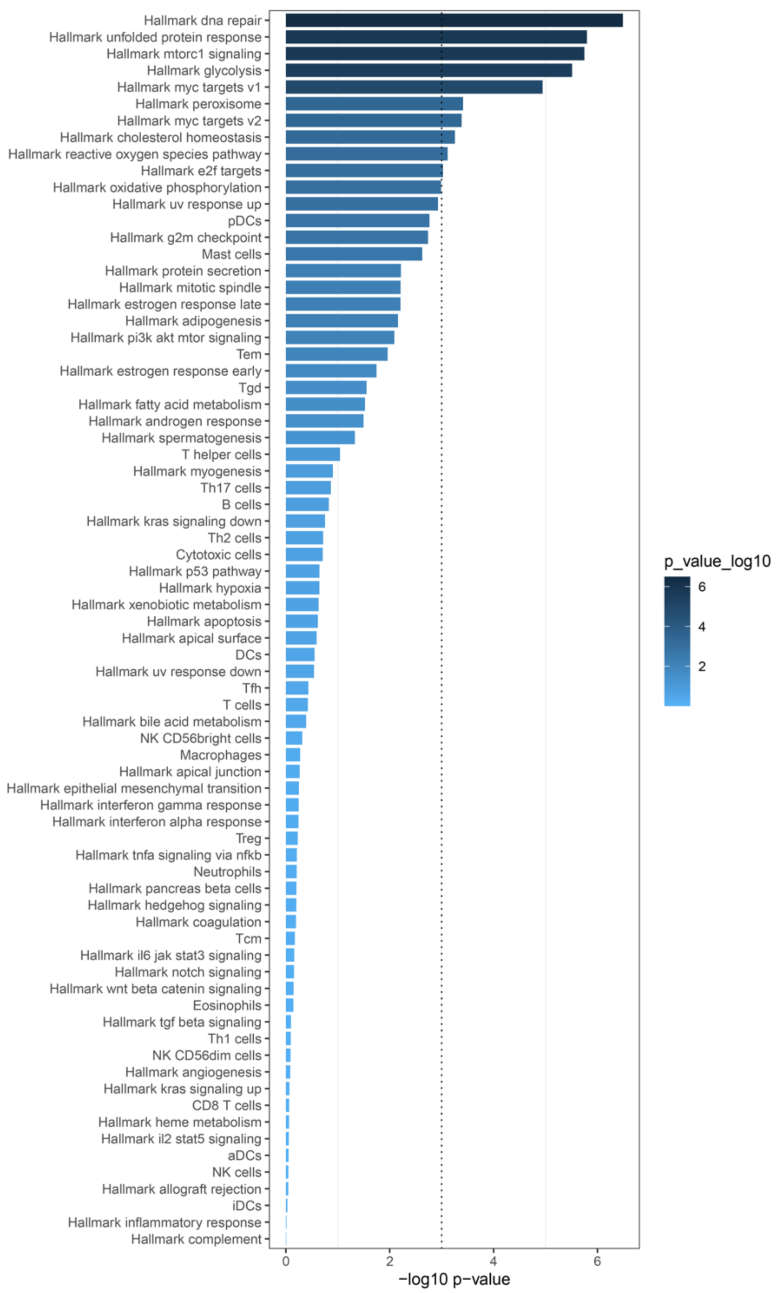

Supplemental Figure S4. Bar plot showing the significant and non-significant up- or down-regulated pathways between pre- and post-imatinib biopsies ranked according to significance (min-log10 p-values). The geneset pathways that were used are derived from the MSigDB hallmark geneset collection [3] and the immune compendium signature collection [4].
